# Dose reconstruction supports the interpretation of decreased abundance of mammals in the Chernobyl Exclusion Zone

**DOI:** 10.1038/s41598-020-70699-3

**Published:** 2020-08-21

**Authors:** Karine Beaugelin-Seiller, Jacqueline Garnier-Laplace, Claire Della-Vedova, Jean-Michel Métivier, Hugo Lepage, Timothy A. Mousseau, Anders Pape Møller

**Affiliations:** 1 Institut de Radioprotection et de Sûreté Nucléaire, Pôle Santé Environnement, PSE-ENV/SRTE, Cadarache, Bâtiment 183, BP3, 13115 Saint Paul lez Durance Cedex, France; 2 Institut de Radioprotection et de Sûreté Nucléaire, Pôle Santé Environnement, PSE-ENV, Bâtiment 28, BP 17, 92262 Fontenay-aux-Roses Cedex, France; 3 Institut de Radioprotection et de Sûreté Nucléaire, Pôle Santé Environnement, PSE-ENV/SEREN, Cadarache, Bâtiment 153, BP3, 13115 Saint Paul lez Durance Cedex, France; 4 Department of Biological Sciences, University of South Carolina, Columbia, SC 29208 USA; 5 Laboratoire d’Ecologie, Systématique et Evolution, CNRS UMR 8079, Université Paris-Sud, Bâtiment 362, 91405 Orsay Cedex, France

**Keywords:** Biodiversity, Community ecology, Environmental impact

## Abstract

We re-analyzed field data concerning potential effects of ionizing radiation on the abundance of mammals collected in the Chernobyl Exclusion Zone (CEZ) to interpret these findings from current knowledge of radiological dose–response relationships, here mammal response in terms of abundance. In line with recent work at Fukushima, and exploiting a census conducted in February 2009 in the CEZ, we reconstructed the radiological dose for 12 species of mammals observed at 161 sites. We used this new information rather than the measured ambient dose rate (from 0.0146 to 225 µGy h^−1^) to statistically analyze the variation in abundance for all observed species as established from tracks in the snow in previous field studies. All available knowledge related to relevant confounding factors was considered in this re-analysis. This more realistic approach led us to establish a correlation between changes in mammal abundance with both the time elapsed since the last snowfall and the dose rate to which they were exposed. This relationship was also observed when distinguishing prey from predators. The dose rates resulting from our re-analysis are in agreement with exposure levels reported in the literature as likely to induce physiological disorders in mammals that could explain the decrease in their abundance in the CEZ. Our results contribute to informing the Weight of Evidence approach to demonstrate effects on wildlife resulting from its field exposure to ionizing radiation.

## Introduction

Thirty years after the accident at the Chernobyl nuclear power plant (Ch-NPP), the subject of its consequences for wildlife living in the Exclusion Zone (the so-called CEZ, that is an approximately 30 km diameter evacuated area surrounding the NPP) is still hotly debated between scientists arguing it had a negative ecological impact^1^ and proponents of no-effects, or even some collateral consequences such as human evacuation being beneficial for the fauna^2,3^. However, whatever their conclusions, these studies do not sufficiently often analyze the underlying data with respect to the most relevant indicator of total exposure when attempting to quantify the dose–response relationship that characterizes the effects of animal exposure to ionizing radiation. As explained by Beresford et al.^4^ simplistic measurements of exposure, such as ambient dose rates or soil concentration activities, cannot encompass all the complexity of actual exposure of wildlife. Contributions of internal and external irradiation pathways to the total dose rates have both to be considered, and this balance depends on radionuclides (type and energy of emitted radiation) and on animal species (age, diet, habitat, use of the environment). Largely used for humans, the dose reconstruction process would also allow the accurate characterization of wildlife exposure required to interpret it in terms of effect. It should be acknowledged that this approach incorporates larger uncertainties than for humans as it deals with interspecific variation in addition to inter-individual differences. However, it is an unavoidable step for a correct analysis of the dose–response relationship.

**Figure 1:**
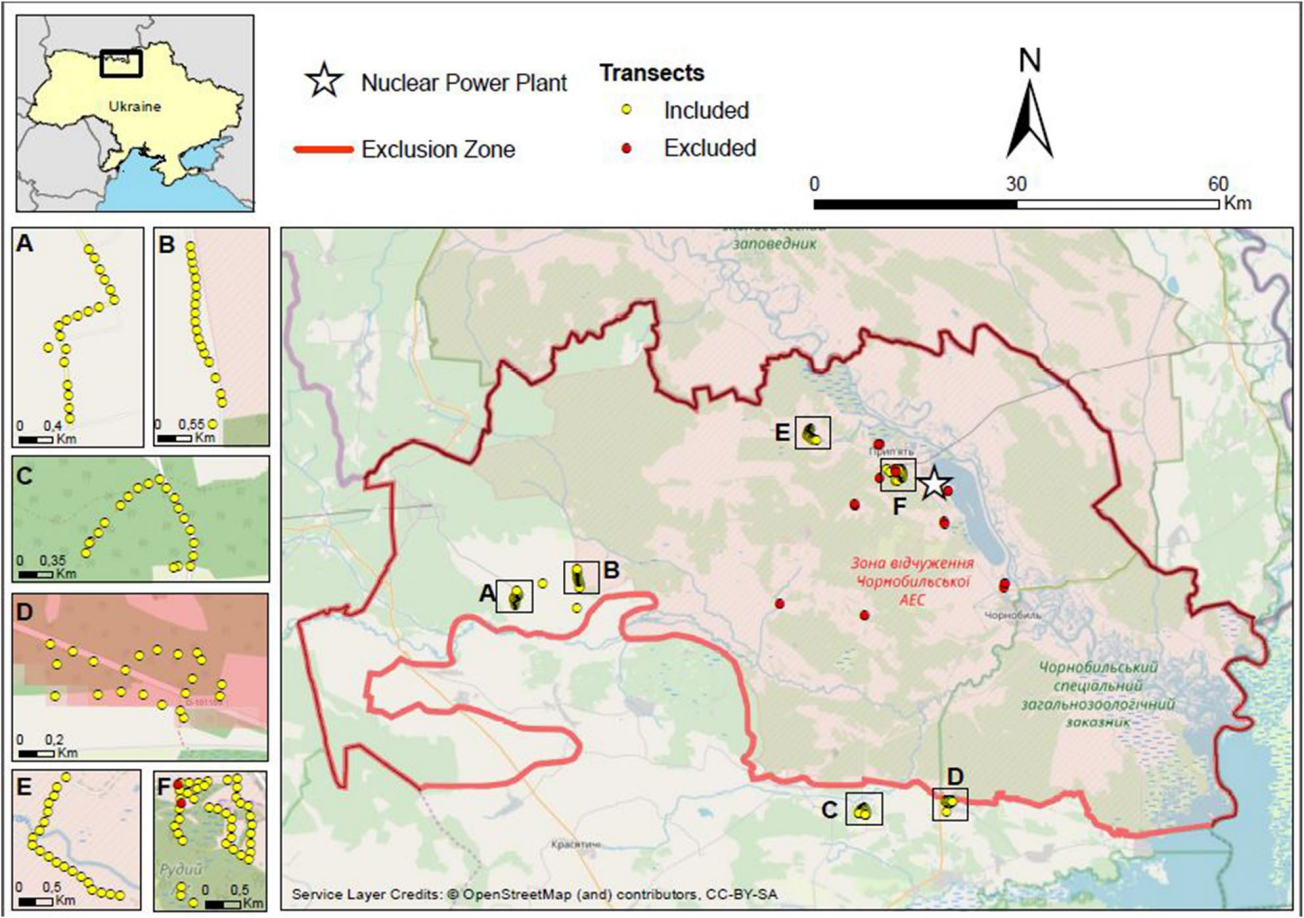
Location of the study sites in or near the CEZ. (Adapted from Møller and Mousseau^1^, and Open Street Map—circles: first point of each transect; red/yellow: excluded/re-analyzed, red line: limits of the CEZ).

Applying concepts inspired from those previously defined for studying the abundance of breeding birds at Fukushima in relation to their chronic radiation exposure^5^, we revisited the results of a field census of mammals in the CEZ^1^, where tracks in the snow were counted along line transects in 2009. The study sites were located west of the Ch-NPP, inside or very close to the CEZ, over an area of 800 km^2^ (Fig. 1). The abundance of mammals was estimated from tracks identified during a single short period, to limit confusion due to multiple sightings of the same individual or deterioration of footprints over time. A number of environmental variables were simultaneously recorded (time of observation, time since snowfall and soil cover type—% of tree, bush, grass), that have to be considered as potentially confounding variables in any statistical analysis of the dose-abundance relationship. Simultaneously, the background radiation level was recorded at the beginning of each transect at which coordinates were obtained from a GPS. The previous analysis of this dataset^1^ emphasized the negative effects of level of ambient radiation on the abundance of mammals and predator–prey interactions in the CEZ. However, two more recent studies^2,3^ found no evidence of such effects on mammal communities in the CEZ or neighboring areas.

Combining life history traits describing the mammals species located by Møller and Mousseau^1^ in the CEZ with their abundance as obtained from the census, we aimed at analyzing in a more rigorous manner the most meaningful dose indicator with regard to the quantification of the dose-abundance relationship characterizing organismal response to ionizing radiation if any^4–7^. We reassessed the dataset by substituting the ambient radiation level used previously^1^ with the total dose (and dose rates) that we reconstructed for individual mammals. We assessed both the external and internal components of the dose (rate) on the basis of (i) the simplified morphological and ecological characteristics of the mammals of interest (Tables S1–S4), and (ii) the radioisotope concentrations in soils, crossing GPS information with the most relevant available data describing soil contamination. We re-analyzed the abundance data considering the reconstructed total dose as an explanatory variable for mammal individuals, with the same environmental descriptors as confounding variables. Optimization of the statistical methods applicable to this dataset led to grouping of data according to two hierarchical taxonomic levels of description. The first analysis focused on all identified mammals, with an additional analysis of “predators” and “prey”. This re-analysis confirmed the results previously obtained^1^, which concluded a relationship between exposure to ionizing radiation and the abundance of mammals. The added value of our work consisted first of assessing a realistic value of the dose absorbed by exposed mammals, applying recognized and fully traceable methods. Second, we checked the consistency of our results with the state of the art with regard to dose (rate)–effect relationships.

## Results

### Total dose over generation time

**Figure 2:**
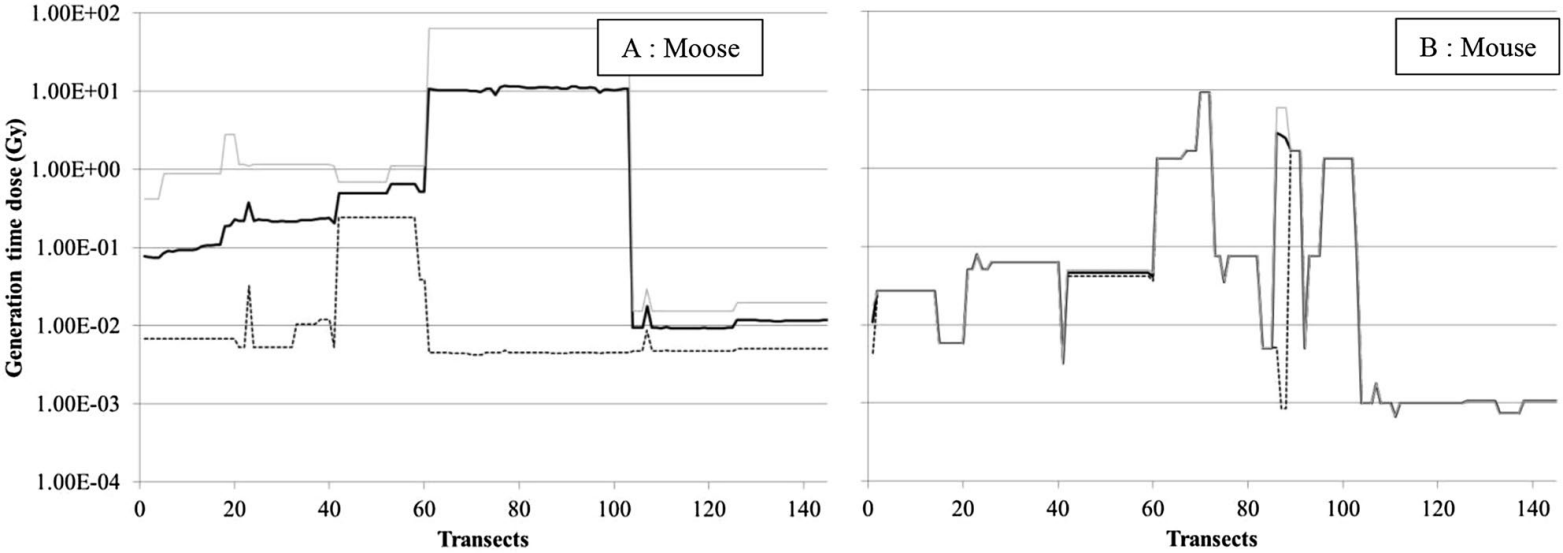
Spatial change of the total dose absorbed by moose and mice over their generation time (result by transect, calculated with minimal (dotted line), mean (black line) and maximal (grey line) activity concentrations of ^137^Cs and ^90^Sr in soil for the given transect).

From soil activity concentrations per radionuclide (see “Methods”, Soil contamination data), we assessed for each mammal species the extreme and mean total dose it absorbed during its generation time (*td*_*j*_). Depending on a species and its home range, basic data were more or less complete. We compared the *td*_*j*_ values for the two mammals having the greatest difference in generation time (Mouse: 627 days; moose: 3,722 days, Table S1). We reported on the same graph (Fig. 2) their minimal, mean and maximal *td*_*j*_ values for each dosimetry area (the potential exposure area centered on the transect origin, see “Methods”), identified by the transect number. The spatial evolution of these *td*_*j*_ datasets presented opposing patterns. We obtained three significantly separated curves (Fig. 2A) for the large mammal along the 145 re-analyzed transects. Its larger home range resulted in a dosimetry area covering sometimes orders of magnitude of variation in the soil activity concentration, due to the so-called “leopard spot” contamination pattern. In contrast, the much smaller home range of the mouse resulted in a quasi-absence of variation in soil activity concentrations, leading to quite similar dose–response curves across the whole study area (Fig. 2B). To make the analysis consistent for all species, we decided to continue only with the mean *td*_*j*_.

**Figure 3:**
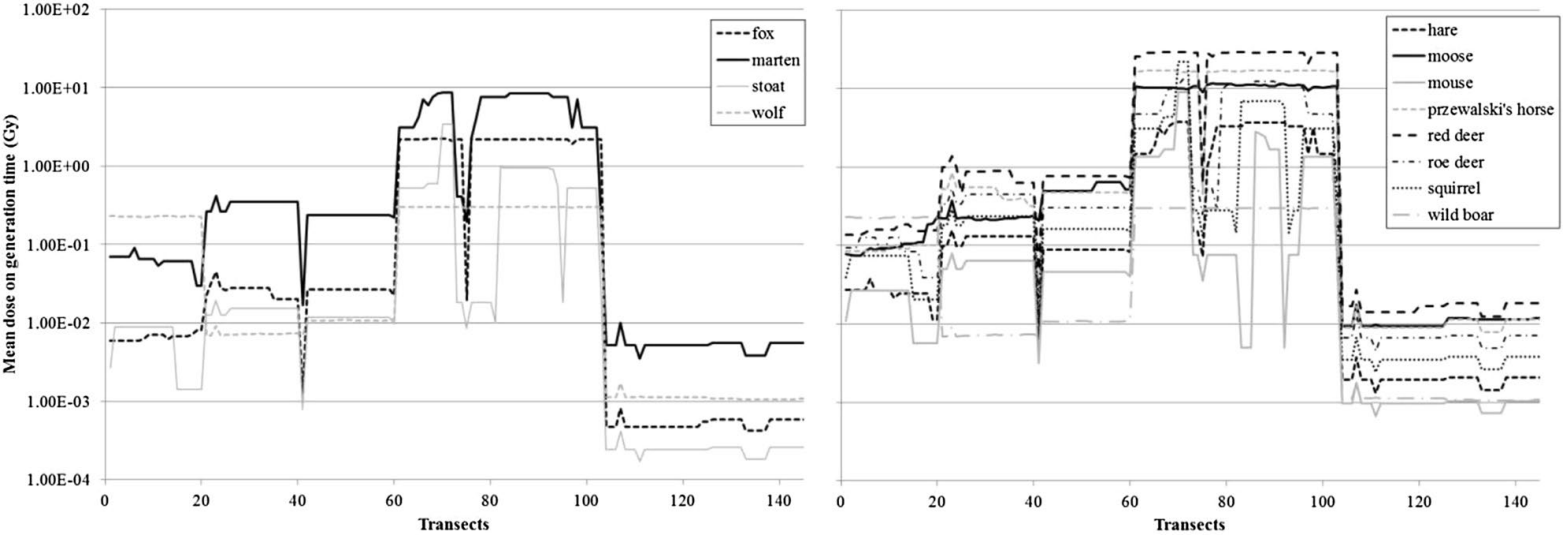
Total dose absorbed over generation time by mammal species as a function of the transect, calculated using the mean activity concentrations of ^137^Cs and ^90^Sr in soil (left: predators; right: prey).

The *td*_*j*_ values varied over five orders of magnitude when considering all species and all transects (< 3 orders of magnitude within a species, Fig. 3), with a clear spatial distinction between groups of transects. The transects close to Pripyat (n° 21 to 60—area F Fig. 1) presented the highest total doses over the generation time (about 10 Gy), ca two orders of magnitude higher than those assessed for the north west of the city (transects 1 to 20—area E Fig. 1). Transects 61 to 103 showed an order of magnitude lower *td*_*j*_ (areas A and B Fig. 1). The lowest total doses, from 10^–3^ to 10^–2^ Gy, were estimated for the transects outside the CEZ (numbered from 104 to 145—areas C and D Fig. 1). Predators experienced total doses from 10^–2^ to 10^1^ Gy in the CEZ, vs. 10^–3^ to 10^–2^ Gy outside (Fig. 3, left). Prey experienced slightly higher values (Fig. 3, right). Predictably, species with the smaller home ranges displayed more spatial variation in doses than species with larger home ranges.

**Figure 4:**
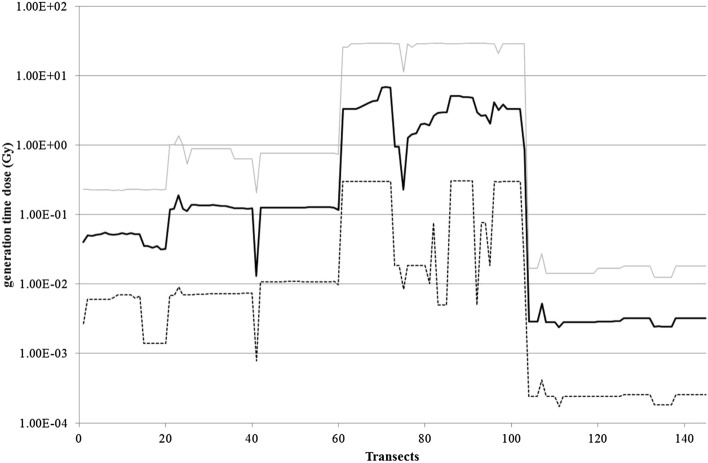
Generation time dose per transect (dotted and gray lines: extreme values among the 12 species; black line: geometric mean on the 12 species- *TTD*).

Our final goal was to assess how animal abundance varied with the *td*_*j*_ values. For this analysis, a single value per transect was needed. However, given that most statistical approaches are intolerant to high frequencies of zeros in the dataset, there was a need to find an approach that limited the number of zeros in the series to be treated. One approach to this problem is to group data into larger categories. Here, for the first level of analysis, all species were grouped into a single category. Since *td*_*j*_ values varied considerably among species for a given transect in a ratio of up to ca 6,000 (Fig. 4), the geometric mean was used as the best solution and the theoretical Transect Total Dose *TTD* was assessed for each transect (see “Methods”, Dose reconstruction). The four-zone pattern previously observed for the spatial evolution of the *td*_*j*_ values per species was well reproduced, attesting to the good representativeness of this parameter with regard to our objective. The *TTD* varied from 1.3 × 10^–2^ to 6.8 Gy inside the CEZ, and from 2.4 × 10^–3^ to 5.2 × 10^–3^ Gy outside.

### Variation in mammal abundance with TTD

**Figure 5:**
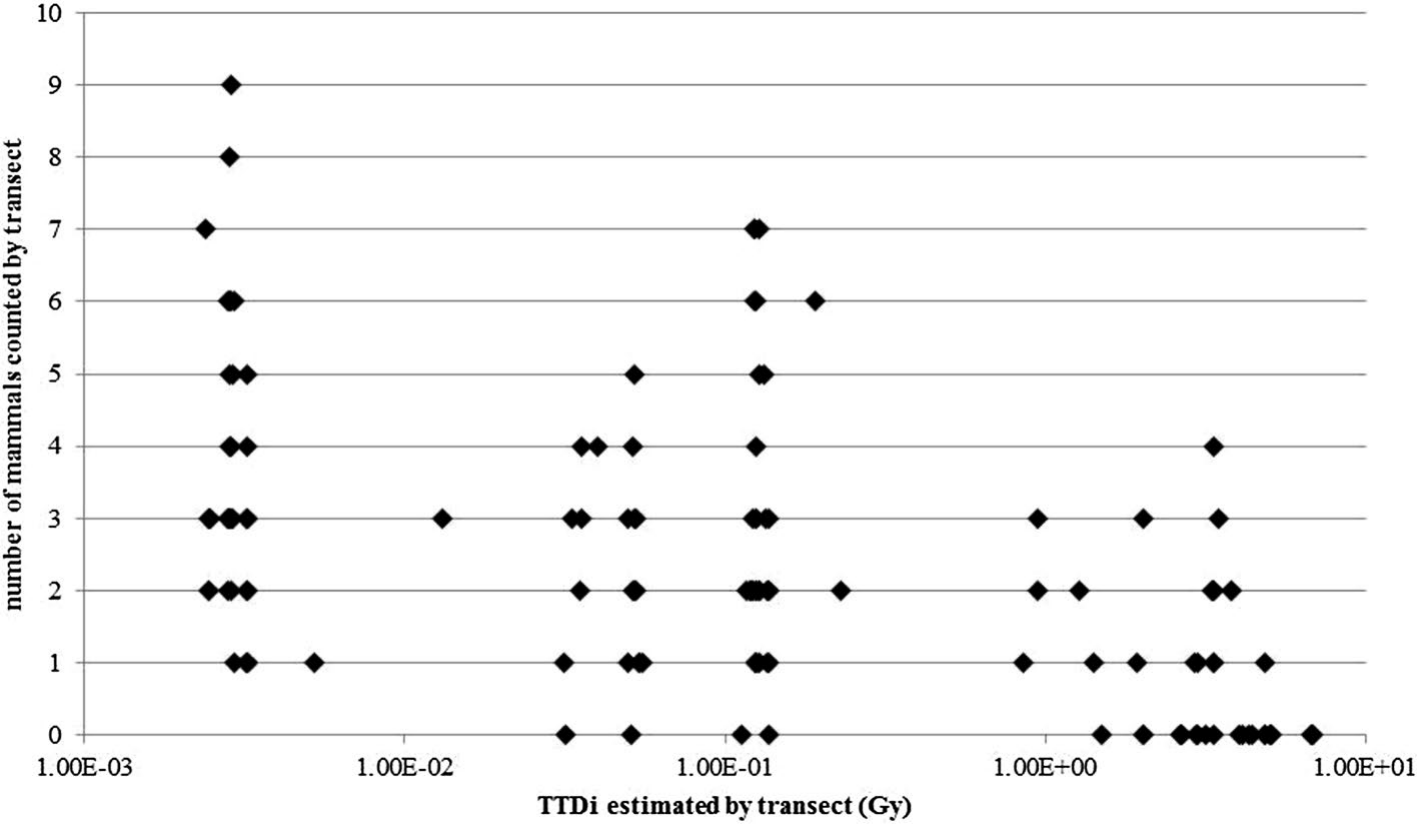
Variation in the number of mammals counted by transect as a function of *TTD*_*i*_.

There was a general trend for decreasing mammal abundance with increasing *TTD* values (Fig. 5), although a scatter of points was a strong indication for the need of a deeper investigation that also considered additional variables known to potentially affect abundance (see SI).

The possible confounding variables included as fixed effects in the Generalized Linear Mixed Models (GLMM) were identified as follows: *Time since snowfall* ([26–52], mean = 35.1 h), (*Time since snow*)^2^, *Bush cover* ([0–90], mean = 22.93%), and *Grass cover* ([0–95], mean = 15%).

The first GLMM fitted expressed variation in mammal abundance with increase in *TTD*_*i*_, including the four possible confounding variables previously identified. The relationship between animal abundance and the *TTD* is significantly negative (Table 1), that is to say, the number of mammals decreased with increasing dose. The analysis revealed that time^2^ (time × time) since last snowfall also explained a significant proportion of the number of mammals observed along a transect. The three remaining factors had no influence on the mammal abundance.

**Table 1:** Regression table for the four tested GLMMs, as a function of the animal categorization and the confounding variables considered.

Animal category	Predictors	Estimate	SE	P value	Adjusted P value
All mammals	TTD_i_	− 1.038	0.150	< 0.001*	< 0.001*
	Time since snow	0.265	0.203	0.192	0.588
	(Time since snow)^2^	− 0.879	0.274	0.001*	0.006*
	Bush cover	− 0.094	0.061	0.122	0.422
	Grass cover	− 0.060	0.063	0.340	0.820
Prey	TTD_i_	− 1.274	0.247	< 0.001*	< 0.001*
	Time since snow	− 0.652	0.190	0.001*	0.003*
	Bush cover	− 0.089	0.111	0.425	0.900
	Grass cover	− 0.157	0.115	0.171	0.538
Predators	TTD_i_	− 0.778	0.185	< 0.001*	< 0.001*
	Time since snow	0.711	0.284	0.012	0.053
	(Time since snow)^2^	− 1.151	0.376	0.002*	0.010*
	Bush cover	− 0.145	0.080	0.072	0.274
	Grass cover	− 0.026	0.088	0.763	0.998
Predators, adjusting for prey	TTD_i_	− 0.798	0.194	< 0.001*	< 0.001*
	No of prey	− 0.017	0.051	0.739	0.999
	Time since snow	0.718	0.285	0.012*	0.063
	Time since snow^2^	− 1.170	0.381	0.002*	0.012*
	Bush cover	− 0.144	0.080	0.073	0.324
	Grass cover	− 0.026	0.088	0.767	1.000

*Significant at α < 0.05.

### Effects of subgroups

The next step of our analysis consisted of exploring ways to refine the analysis per subgroup of mammals. Following the example of Møller and Mousseau^1^, we first distinguished prey and predators, applying the same approach as for the entire mammal category. The number of prey was significantly negatively influenced by *TTD*_*i*_ and positively affected by *time since snowfall* (Table 1). The number of predators was also negatively related to *TTDi*, and it was marginally positively influenced by *Time since snowfall*, and significantly negatively related to *(Time since snowfall)*^2^. Adding the number of prey as a potential new confounding variable did not add significantly to the data analysis. The robustness of this first category of analysis is already strongly constrained by the small numbers of prey and predators per transect. Additionally, the grouping of species into prey and predators was arbitrary based on their diet, considering carnivorous and omnivorous mammals as potential predators and other mammals as potential prey. The reality of the prey-predator relationship between the species of interest has not been considered (small carnivorous such as stoat does not feed on large mammals). According to this well identified limitation of the number of individuals, we decided not to investigate finer scale divisions among taxa as frequencies for most species were too low to permit a robust statistical analysis.

## Discussion

There has been considerable discussion and some disagreement concerning the possible effects of low to moderate dose of ionizing radiation on natural populations^2,3,8–12^. However, most investigators agree that the poor estimate of actual levels of exposures in terms of doses to wildlife is one of the highly relevant explanations that could underlie differences in opinions concerning radiation effects on wildlife^4,5,13,14^. This led to recommendations with regard to the nature of data to acquire when conducting field studies^5,15^. Suggested good practices, such as more thoughtful sampling protocols, should apply to future studies. However, it would be wasteful to not better value existing data as often as possible, through their re-analysis and reinterpretation if and when needed. The current study clearly illustrates the value of such an approach. By proposing such re-interpretations, there are inevitable issues related to data availability, assumptions to be made, and additional uncertainties. Of particular note, is that a robust analysis of field data related to ionizing radiation requires the most accurate dose assessment possible^5,14,16^. When re-analyzing past studies, this implies the implementation of a dose reconstruction that combines ecological and radiological aspects. The present study required that we adapt the available historical data to our spatial and temporal needs in order to assess soil contamination levels. Generic life history and morphological information were collected for all mammal species, ignoring their potential site-specific characteristics. From this, we made the best use of the initial dataset and the additional information that we were able to gather.

Due to limitations in the initial dataset and constraints imposed by statistical models, we were only able to robustly analyze patterns of variation for the entire mammal assemblage, and predators and their prey. Because of the rarity of many species, there was insufficient variation among sites to permit a more detailed analysis at any lower taxonomic level. Future studies should endeavor to increase overall sample sizes as well as promote the development of new statistical models better able to accommodate sparse data.

The first result of this re-analysis of the dataset collected by Møller and Mousseau^1^ confirmed their first conclusion. There is a correlation between the increasing exposure to ionizing radiation of mammals due to radionuclides emitted during the NPP accident and the decrease in their abundance. In terms of dose rates, the range of the lowest values across all sites varied from 6.6 × 10^–3^ µGy h^−1^ (stoat outside the CEZ) to ca 10^–1^ µGy h^−1^ (wild boar outside the CEZ). At the opposite end, the highest dose rates ranged from 4.4 µGy h^−1^ (wolf, West of the CEZ) to 6.1 × 10^2^ µGy h^−1^ (mouse, West of the CEZ). On average across the 145 transects, the highest dose rate was recorded for the Przewalski’s horse (7.0–10^1^ µGy h^-1^), and the lowest for the wolf (1.8 µGy h^−1^). These values were calculated for an average individual of each species, which is supposed to be representative for the mean exposure of populations of this species. The area on which the dose rates were assessed was the species home range, that is to say the area sufficient for individuals of this species to carry out their normal functions. This area is also considered of relevance to the maintenance of populations^17^.

Natural background exposure dose rates would constitute a relevant point of reference to put in context the additional dose rates we assessed. Their characterization is scarce, and spatially limited. Some data have been reported for terrestrial wildlife in parts of the UK^18^. For mammalian reference animals (RAPs)^19^, the range of background exposure varied from 5.0 × 10^–2^ to 1.4 × 10^–1^ µGy h^–1^. This corresponds to the lowest values we estimated outside the CEZ, that is to say two to three orders of magnitude lower than the highest dose rates we calculated for wolf and mouse. These latest values are consistent with other assessments made in the CEZ, for example for small rodents (38 to 61 µGy h^–1^ at the western edge of the Red Forest^20^).

The range of levels of exposure assessed in this paper for populations of mammals largely cover the Derived Consideration Reference Level (DCRL) of 4.2 to 42 µGy h^–1^ adopted by the International Commission on Radiological Protection (ICRP) for mammalian RAPs. Just above this DCRL, defined as the band of dose rate for which there is very low probability of effects occurring to individuals of mammals, the ICRP recognizes a potential for reduce reproductive success which could explain a decrease in abundance of mammals. Additionally, the two ranges of values include the generic screening benchmark defined as “safe” at the ecosystem level during the ERICA project^21^ (10 µGy h^–1^). Looking more precisely at ecosystem sub-levels, vertebrates among which mammals would be protected by respecting a maximal dose rate value of 2 µGy h^–1^ established as a first estimate^22^. Both the 10 µGy h^–1^ and the 2 µGy h^–1^ values were derived on the basis of effects observed at the individual level, applying recognized methods to extrapolate at the population level^21,22^. A look in the FREDERICA database^23^ suggests that the range of dose rates reported here could result in various observable effects on individuals of a variety of mammals including the otter (10 µGy h^–1^: minor decrease in body weight and moderate decrease in population density^24^), mice (16 µGy h^–1^, significant increase in life span^25^) and vole (42 µGy h^–1^, reduction in some blood cells^26^). The actual consequences for population, and a good way to asses them, of such effects and any other effect observed on individuals are an area of intensive research in the field of radiation protection of wildlife. However, the global view obtained from the combination of the whole set of effects data gives an intrinsically consistent panorama which may partly explain the reduction in mammal abundance described in the CEZ by Møller and Mousseau^1^ (Fig. 5).

**Figure 6:**
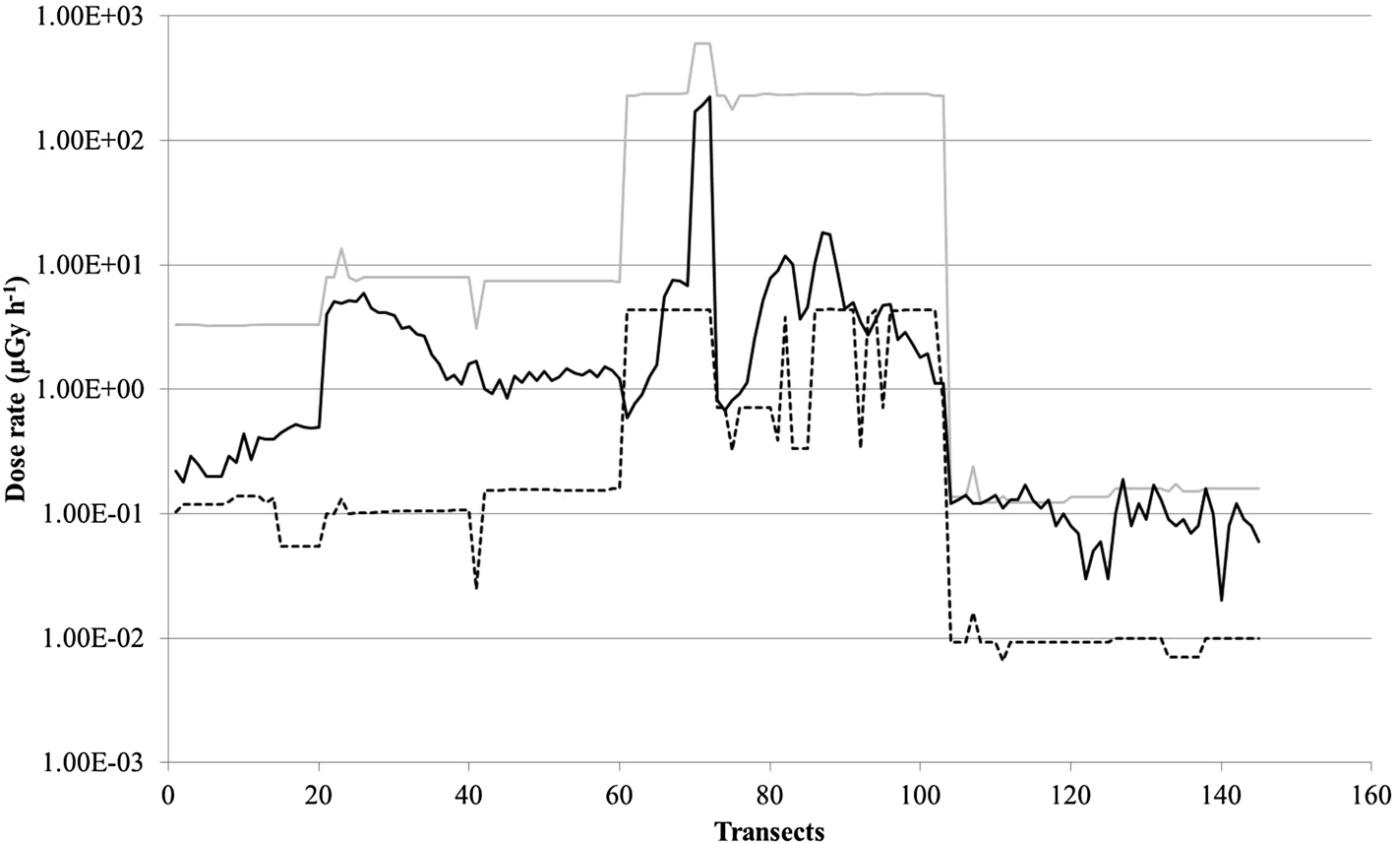
Comparison per transect of the measured ambient dose rate (black line) and the extreme values of dose rate reconstructed for the twelve mammal species (minimal: dot line; maximal: grey line).

Our comprehensive dose reconstruction generates a re-assessment of the levels of exposure of mammals in the CEZ that are now numerically comparable with those cited in the literature as potentially deleterious. This process leads to dose rate estimates potentially experienced by the twelve species of mammals due to their exposure to ^137^Cs and ^90^Sr that differ by up to ca. three orders of magnitude on the same transect, and by five orders of magnitude across the whole study area. Use of simple ambient dose rates in such cases led to (i) ignorance of this variation and (ii) to the conclusion that effects could occur at dose rates up to a factor three lower than those actually estimated (10^–2^ to 2.3 × 10^2^ µGy h^−1^ vs. 6.6 × 10^–3^ to 6.1 × 10^2^ µGy h^−1^). The ambient dose rate is in general neither representative of the minimal nor maximal value of the dose rate assessed per transect using our reanalysis (Fig. 6).

In addition to the “dose” effect, re-analysis revealed that one of the confounding variables we were able to consider also affected the number of mammals. Mammal abundance, as well as the number of prey and predators, varied significantly with the variable *Times since snowfall*. More precisely, for mammals as a whole and predators, their abundance firstly increased and then decreased with this time interval. For prey, their number always decreased. The effect of time elapsed since the last snowfall was considered but found not to be as significant in the initial analysis conducted by Møller and Mousseau^1^. The abundance response to this variable was however expected as this is an obvious known weakness of the counting method: the less snow available, the fewer tracks that are visible and identifiable.

Our re-analysis of dose–response relationships in the CEZ provides significant insights concerning the degree of negative impacts on wild mammal populations. For mammals potentially present in the CEZ in 2009, an increase by a factor of 10 of the total dose (*TTD*) was associated with a decrease of ca 60% of the number of all mammals, 66% of the number of prey, and about 50% of the number of predators (Table 2). Our analysis found no evidence for a direct effect of number of prey on predator abundance, probably because of the small number of animals.

**Table 2:** Decrease in individual number by animal categories with an increase by a factor 10 in the generation time dose.

	Estimates of *TTD* predictor^a^	Decrease rate (1 − e^−β^)	
		Value (%)	Confidence interval 95%
Mammals	− 0.889	58.9	47.3–67.9
Prey	− 1.091	66.4	49.2–77.8
Predators	− 0.666	48.6	30.0–62.3
Predators (number of prey as cofounding variable)	− 0.683	49.5	30.1–63.5

^a^log_10_ transformed but not scaled (i.e. not centered and not reduced), *TTD* in Gy before log transformation.

## Methods

The principle of dose reconstruction supposes to gather a significant amount of data in multiple areas, from radiological measurements to ecological information for each species. Since our primary dataset was not acquired with this objective in mind, we faced a lack of information for some descriptors. We filled these missing values using reasonable assumptions founded on scientific justification as described below.

### Study sites and mammals tracks

**Figure 7:**
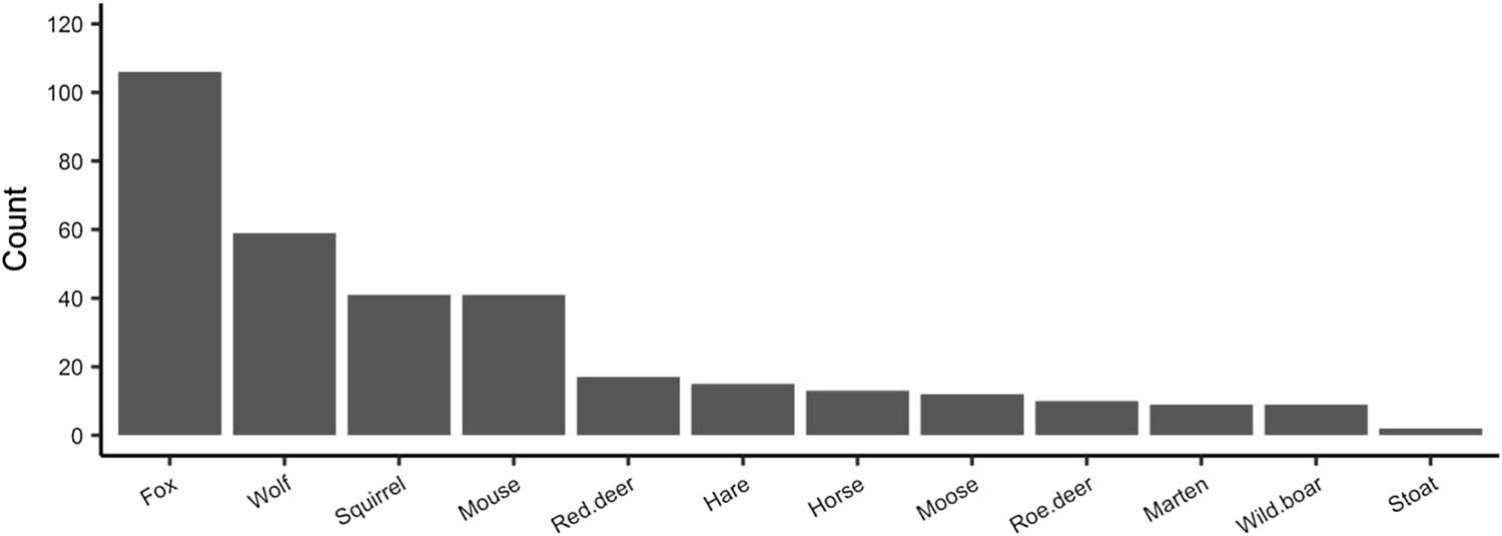
Count numbers per mammal species over the 145 studied transects.

We re-analyzed the dataset described by Møller and Mousseau^1^. They used foot prints following fresh snow fall to estimate abundances of mammals, as counted by a single observer on a single ca. 2 day period of 3–4 February 2009. A total of 161 line transects were surveyed, each with a length of 100 m. Transects were separated by at least 50 m (but usually 100–500 m), and were located along roadsides (Fig. 1). A rigorous examination of the consistency and homogeneity of the dataset led us to exclude 16 transects from our analysis. Sixteen of these transects (see SI) were investigated during a different period (January 21 or February 17 and 18, 2009), applying a different experimental logic. They did not belong to the same sampling plan, and their use would have reduced the statistical significance of our analysis. This reduction did not affect the diversity of mammal species observed. The revised dataset included the abundance of 12 species of mammals distributed over 145 transects (Fig. 7). Foxes were the most frequently observed species, followed by wolves. Large prey (deer, horse, moose, and wild boar) had count numbers from about ten to twenty individuals.

**Figure 8:**
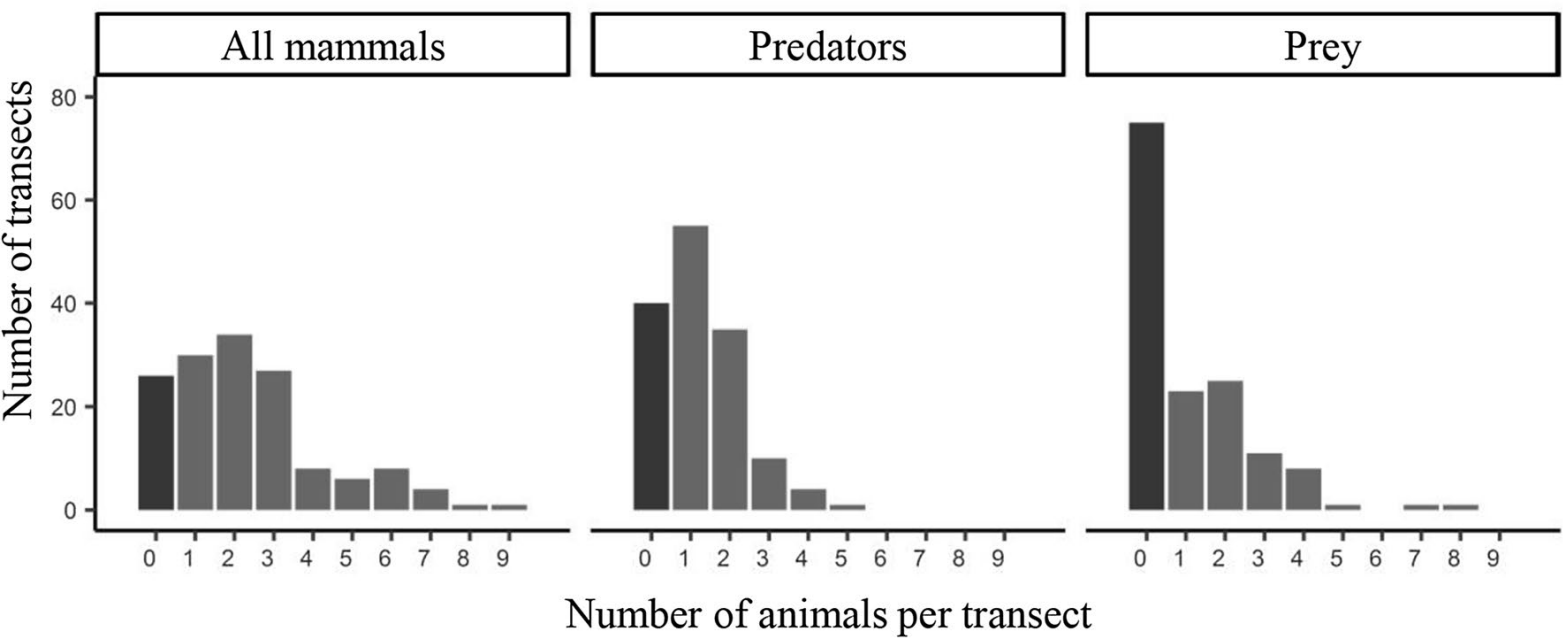
Number of transects with a given count of individuals per category of animals.

About 18% of the transects were devoid of mammal activity during the study period (Fig. 8). Predators tended to be more widely distributed than prey, being observed in 72% of transects while prey were not seen in about half the transects. Most transects (> 80%) had fewer than 4 individuals. The paucity of observations prevented a more refined analysis of the taxonomic structure of these data.

We used a number of additional descriptors for each site as covariates in our analysis, as described by Møller and Mousseau^1^. These included temporal descriptors (i.e. time of day the counting started, time since the last snow fall) and environmental factors (percentage cover with grass, bushes and trees to the nearest 5%). Ambient radiation levels were determined from averaged repeated measurements (2–3) at ground level with a hand-held Geiger counter (Model: Inspector, SE International, Inc., Summertown, TN, USA).

### Dose reconstruction

The principles for reconstruction of total radiological doses absorbed by animals were described in detail in a previous similar study of dose reconstruction for birds in the Fukushima area^5^ (SI). According to this method, we estimated for the mammal species *j* and the radionuclide *r* the external and internal dose rates (respectively *EDR(j,r)* and *IDR(j,r)*; µGy h^−1^, see supporting Excel® file) absorbed by mammals of a given species. We used radionuclide activity concentrations measured in soils and calculated for animals, multiplied by the ad-hoc Dose Coefficients (DCs, Table S8). These coefficients, specific for the combination of radionuclides, species and irradiation pathway, were determined per radionuclide and daughter element(s) for adult stage of each species with the EDEN v3.1 software^27^.

The internal and external irradiation dose rates absorbed by mammal species were calculated according to the equations described elsewhere^5^ (and recalled in SI). The total dose rate absorbed by the mammal species *j*, *TDR*_*j*_, is the sum of the internal and external dose rates assessed for each radionuclide, applied to all radionuclides of interest (SI Excel® file).

We assessed the total dose to a given mammal species *j* considering that adult individuals are exposed to ionizing radiation at a constant dose rate during a period that corresponds to the generation time *L*_*Gj*_ of the species (average age of parents of the current cohort reflecting the turnover rate of breeding individuals in a population; SI). Consequently, the total dose absorbed by each species *j* at a given transect *i* noted *td*_*j,i*_ resulted from

1 $$ td_{j,i} = TDR_{j,i} {\text{x}}L_{Gj} $$

This total dose was calculated for each species and each transect, whether the presence of the species on this given transect has been confirmed or not. This is the theoretical total dose the animal would receive if living there.

The final objective of the re-analysis was to study how abundance of different groups of mammals (all mammals, prey and predators) identified at the study sites vary according to their exposure to ionizing radiation. We needed a unique value of dose per transect, representative of the average exposure of all animals indirectly observed along this transect. The *td*_*j,i*_ values presented large ranges of variation (intra-transect ratio from ca. 30—transect n°10—to 6,000—transects n°83, 84 and 92). Their geometric mean for all species on a given transect *i* was calculated as the most relevant indicator, named Transect Total Dose and abbreviated *TTD*_*i*_. Calculating the mean on all species, whatever they have been counted on the transect or not, gives a highly representative estimate of the level of exposure on the considered transect, not of the exposure of the counted species. This was also justified because we considered the zero count as relevant information. Such a number may have two origins: either the species has never occupied the surroundings of the transect (e.g. area not suitable for its needs) or it has disappeared. In both cases, the series of possible confounding variables considered in our statistical analyses will allow us to include this information (see “Methods”, Statistics).We used the geometric mean value in order to limit the influence of extreme values on the results^28^.

### Mammal species and associated assumptions

We deliberately chose to limit our analysis to adults in order to minimize the assumptions required to achieve our calculations. It is generally recognized that juveniles may be more sensitive to exposure to pollutants than adults. Juvenile development and growth mobilize resources that are no longer available for their protection. Juveniles differ from adults in their diet, behavior and physiological characteristics. Moreover, these characteristics change with time from birth to maturity. Such changes can have large implications in terms of dose reconstruction and associated uncertainties. Thus it is necessary to identify periods of development during which individual characteristics can be considered constant, and to be able to collect data corresponding to the needs for dose reconstruction. This approach is possible for a single species, but would be much more speculative for all 12 of our species of interest. Since our understanding of adult life history is likely to be more robust than that of juveniles for the purposes of dose reconstruction, we have ignored juvenile stages for this analysis. Moreover, time from birth to maturity is generally short with regard to generation time (Table S1), and discounting the corresponding contribution to the total dose would underestimate its actual value in a way that makes our results an acceptable proxy for the quantification of the response of mammals to their exposure to ionizing radiation.

For each of the 12 species under consideration, DC calculation required us to simplify the representation of adults as ellipsoids of known mass and size (geometric characteristics, Tables S1 and S2), and to define media elementary composition (Table S3). In the same way, a basic animal life style was described considering the time spent (i) in a burrow if relevant for the species and (ii) standing or lying on soil for all species (Table S4). Finally, as much attention as possible was paid to the species’ diet (omnivorous, carnivorous or herbivorous) to select the most appropriate value for the concentration ratios (CR) required to quantify the radionuclide aggregated transfer from soil to the animal (Table S5). When available, site-specific CRs were preferentially used, to reduce the large uncertainty associated with the choice of a CR value. This uncertainty is a well-known weakness of the assessment of activity concentrations in animals applying the equilibrium approach^29,30^. By default for site-specific data, the choice was made to refer to best-estimates published in an international compilation of data^31^. All data depending on the nature of the radionuclide were collected or calculated for the elements Cs and Sr and their isotopes present in the accidental releases for the Chernobyl NPP accident (Table S6). Since dose (rate) is additive in terms of the resulting effects of exposure to ionizing radiation, it is essential to exhaustively characterize the source of radioactivity under examination in terms of quality and quantity.

### Soil contamination data

We conducted two preliminary studies to streamline and optimize data collection, and to limit the assumptions required to fill potential data gaps. First, we explored the depth of contaminated soil for consideration in the calculation of DCs. A potential maximal depth of 10 cm has been reported for the radioisotopes characteristic of the accident fallout (Table S6), which is in agreement with observed and predicted contamination profiles for ^137^Cs (Fig. S1). In the end we used a 20 cm layer, in a conservative but realistic way, as increasing reasonably the soil depth increases the amount of radioactivity to which mammals could be exposed. Despite the much larger original spectrum of radionuclides, it is largely assumed that today both ^137^Cs and ^90^Sr should be the main markers of the impact on the environment of the NPP accident, due to the emitted quantities and their radioactive half-lives. Radionuclides contribute very differently to the total dose absorbed by animals depending on the energies and nature of their emissions^32^. We thus secondly investigated the role of the 10 radioisotopes for which we found activity concentrations in soil considering their realistic extreme values in a given location (Table S7). We assessed the corresponding total dose rates on one hand for the pair ^137^Cs + ^90^Sr and their daughters, and on the other hand for the remaining radionuclides, for two contrasting mammal species, a small carnivore and a large herbivore (Fig. S2). Whatever the scenario, the dose rate due to ^137^Cs and ^90^Sr represents at least 94% of the total exposure. We assumed that other radionuclides can be ignored without significantly skewing our results, taking into account all the associated uncertainties. This considerably limits the data search and collection (focused on selected isotopes, e.g. DC values, Table S8) as well as the assumptions necessary to achieve the dose reconstruction. It results in a reduction of calculations needed but also of conservatism of the approach, while keeping it at a level sufficient for our needs.

**Figure 9:**
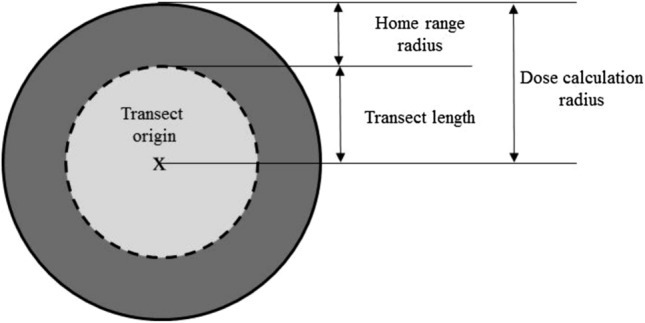
Definition by species of its potential exposure area from which the dose (rate) is calculated.

Measurements of soil radionuclide activity concentrations have been extensively conducted in the CEZ and around since the accident. To best cover the spatial and temporal scales of our study, we combined different data sources^6,33–35^ (plus the REDAC database, V. Kashparov, personal communication). ^137^Cs and ^90^Sr soil activities were assessed for each transect. We took into account both the transect length (100 m) and the species home range (Table S1) to define a potential exposure area for each species present on a given transect (dosimetry area, Table S2). This circular area is centered on the transect origin, located by its GPS coordinates, with a radius of 100 m (transect length) plus the radius of the species’ home range (Fig. 9). Using GIS, we crossed referenced this information with the geo-located contamination data from all the references identified. When several measurements were available for the same dosimetry area, we retained their extreme and mean values (i.e. in general three different values per dosimetry area). When only one single measurement was available, we used these data for both extreme and mean values. When no data were available in a given area, soil activity was assumed equal to the one measured at the nearest soil sampling point. The radioactive decay occurring during the period of dose reconstruction (i.e. the generation time) was ignored with regard to the ratio between the generation times (highest value for the red deer *L*_*G*_: 5,210 days, ca. 14 years) and the radionuclide periods (about 30 years for both ^137^Cs and ^90^Sr). This assumption contributes to the conservatism of the approach. The final dataset included three values of soil activity concentration per radionuclide (^137^Cs and ^90^Sr) for each species on each transect (i.e. more than 10,000 values). We arbitrarily decided not to use more complex data treatment such as krieging. Due to the highly heterogenous “leopard skin” pattern of the soil contamination, we considered such approaches not particularly robust as they give an apparent continuity to soil contamination between measured values. Using only actually measured values helped to limit the number of assumptions required by our calculation, already high. We acknowledge however that a spatialized statistical approach to better assess the soil contamination is an interesting perspective to refine the dose reconstruction.

### Uncertainties

The first source of uncertainty in this study was its field protocol, which did not allow screening of the possibility of a double count of the same animal. This is a well-known weakness of such census methods. This “old fashioned” approach of field counting was largely applied in the past, as it is something relatively easy and simple to implement, requiring relatively few resources in contrast to more technological methods. The related and inherent disadvantage is the uncertainty around the count that is difficult to quantify. The way the census was conducted was though to reduce this uncertainty, by exploring somewhat distant transects in a short period of time. This is not a guarantee that individuals have not been counted more than once, but the application of recommended best practices when using such methods.

Other sources of uncertainty appear in such a dose reconstruction, which is a highly uncertain exercise requiring numerous assumptions. The use of CRs was previously acknowledged as a major source of uncertainty that we managed by constraining the value by the diet and using preferentially site-specific values or by default best-estimates such as CR values provided by the IAEA^31^. Using these values under-predicted the transfer of ^137^Cs to predatory versus to prey species^31^. Wood et al.^36^ reported from previous studies that the transfer of cesium to carnivorous species such as those classified in our study as predatory was suggested to be higher than for mammals at lower trophic levels. At the opposite, values summarized by the IAEA showed a significantly higher transfer of cesium to omnivorous and herbivorous mammals, as data in this database relate^31^ mainly to insectivorous small mammals rather than to species similar to our predatory mammals. In the absence of site-specific CR values, the use of best-estimates remained the best option.

We applied this logic to any other ecological or biological parameter required by the dose calculation (home range, animal size, etc.). Our choice was to make an assessment as specific and realistic as possible, without propagating uncertainty characterized at a global scale. For all parameters, there were insufficient local data to characterize their local variation. Soil activities showed rapid spatial change. This is a well-known characteristic of the contamination in the CEZ and it was the only data that we were able to characterize locally in terms of range of variation. We decided to consider only this site-specific uncertainty in our calculations.

A last source of uncertainty in approaches such as the one applied here is the existence of confounding factors. A number of additional variables are known as potentially affecting mammal abundance, such as environmental characteristics or human activities. The first problem consists in identifying these variables, and then to characterize them. What are the necessary and sufficient parameters to collect, when and how? Regarding the description of the environment, the minimal dataset usually acknowledged as relevant has been collected during the census (soil cover, time of observation, time elapsed since the last snowfall). If time data provide objective information, soil cover is observer dependent. This uncertainty was reduced due to observations done by a single observer. This ensured a high comparability between transects. The interaction between animal abundance and human activities is somewhat more complex to characterize. The nature of the CEZ led us to consider only the potential for repulsion of industrial activity linked with the NPP or attraction of farming areas. Characterizing the latest areas was highly uncertain (see dedicated paragraph in SI). There may be a significant time shift between the time of required data were acquired and the time of census. There may be also problems of spatial definition due to labels used in the available sources of information.

### Statistics

The re-analysis of the dataset gave the opportunity to investigate the role of complementary data related to the impact of human activities. Potential spatial interactions between industrial and farming activities, present in the CEZ, and exposure areas of all or parts of the mammals were considered before to be dismissed as non-significant in the conditions of this study (see SI). The set of confounding variables finally retained was the same as for the initial study, that is to say the environmental descriptors that were recorded during the census (time of observation, time since snowfall and soil cover type expressed as % of tree, bush and grass).

All statistical analyses were performed in R^37^. We first tested the variation in mammal abundance with the *TTD*_*i*_ increase through the development of a Generalized Linear Mixed Model (GLMM), assuming Poisson error distribution. The main predictor (*TTD*_*i*_) was log-transformed and then centered on the mean and scaled by the standard deviation. The multicollinearity between possible confounding variables was checked through the Pearson correlation coefficient (omitted if Pearson correlation coefficient > 0.85, and using^38^ a Variance Inflation Factor < 3), in order to include in the non collinear standardized variables in the model. Site was introduced as a random effect, and transects were treated as statistically independent observations. Secondly, the variation in abundance of mammal subgroups (predators and prey) was tested in the same way with regard to the *TTD*_*i*_ increase. The same fixed and random effects were considered. The last analysis aimed to analyze the variation in predator abundance with *TTD*_*i*_, while considering the number of prey as an additional fixed effect.

Finally, four GLMMs were used to test the following hypotheses (where No means number):

2 $$ No \, \;mammals\sim TTD_{i} + \, Time \, \;since\; \, snowfall \, + \, \left( {Time \, \;since\; \, snowfall} \right)^{2} \, + \, Bush\; \, cover + \, Grass \, \;cover + \, (1|Site) $$

3 $$ No \, \;predators\sim TTD_{i} + \, Time\; \, since \, \;snowfall \, + \, \left( {Time\; \, since \, \;snowfall} \right)^{2} \, + \, Bush \, \;cover + \, Grass \, \;cover + \, (1|Site) $$

4 $$ No\; \, prey\sim TTD_{i} + \, Time \, \;since\; \, snowfall \, + \, Bush \, \;cover \, + \, Grass\; \, cover \, + \, (1|Site) $$

5 $$ No\; \, predators\sim TTD_{i} + \, No \, \;prey \, + \, Time \, \;since \, \;\;snowfall \, + \, \left( {Time \, \;since \, \;snowfall} \right)^{2} \, + \, Bush \, \;cover + \, Grass \, \;cover + \, (1|Site) $$

All GLMMs were fitted with the lme4 package^39^. For each model, we computed adjusted p-values using the multcomp package in order to take into account the multiple null hypotheses tested simultaneously^40^.

## Conclusions

The effect of exposure to ionizing radiation for wild mammal populations in the Chernobyl Exclusion zone was examined. Our re-analysis of exposure levels included measures of animal life history and past and contemporary radionuclide levels in order to estimate total doses for individual organisms in a manner not previously conducted. This novel approach led to new insights that suggest that likely doses to some animals were often much higher than those estimated using simple measures of ambient radiation levels with doses consistent with those expected to generate deleterious effects based on conventional radiological protection criteria for ecosystems. Our new analysis suggests that a tenfold increase of the mean dose absorbed by mammals over their generation time corresponds to a decrease in total abundance of about 60%. These findings tend to confirm the linkage established by the initial study, by revising upwards the dose rate responsible for the observed effects. They remain however fragile, due to the many associated uncertainties. Especially, despite our effort to consider as many as possible confounding factors, the difficult characterization of some did not allow their fully satisfactory interpretation.

The process of dose reconstruction has proven necessary and useful, as this revision led to estimated levels of exposure correlated to observed effects on mammals consistent with the available knowledge on the toxicity of ionizing radiation on these animals. In large part, this new analysis resolves some of the conflicts that were mentioned in the introduction. However, dose reconstruction alone is not sufficient to demonstrate a causal relationship between exposure and the observed effects. But, it is a step in the right direction and, when combined with the large spatial scale of the Chernobyl disaster and the ability to survey biological consequences at multiple locations across a regional landscape, one can at least have some confidence that the observations are realistic in the light of current knowledge of radiation effects. Our results should be seen as one line in a larger Weight of Evidence approach to demonstrate in situ the effects of exposure to ionizing radiation on wildlife.

More generally, our re-analysis indicates that existing datasets can be a valuable source of new knowledge regarding effects of exposure to ionizing radiation for wildlife in the field, as long as dose reconstruction is possible. This reconstruction process may be long and complex, and there are unavoidable assumptions required to fill each data gap that may introduce additional uncertainties. However, the current study indicates that it may be productive, constructive and useful to implement modern re-analyses to review initial findings that did not fit “conventional wisdom”.

The findings of this study highlight two general issues that apply to all ecological research. The first issue relates to the care that must be taken in designing and planning field studies. It is crucial to pay sufficient attention to and adequately characterize the environmental conditions under which the study is conducted and to report these additional data as completely as possible when publishing so that subsequent re-analysis might be conducted if needed. The second issue concerns the need for sensitivity and uncertainty analyses in dose reconstruction processes (although this applies to any ecological analysis). It is only by collecting appropriate and relevant qualitative and quantitative information that field observations may be properly interpreted.

## Supplementary information

Supplementary Information 1.

Supplementary Information 2.

Supplementary Information 3.

## Data Availability

Original raw data on mammal abundance from the survey in the CEZ are provided as Supporting Information, with additional descriptors of the environment collected simultaneously. Activity concentrations in soil and animals, dose conversion coefficients and ecological parameters required by the dose reconstruction process are also presented as Supporting Information, as well as the dose rates and doses they permitted to calculate.
