# Supplementary material for: Dose reconstruction supports the interpretation of decreased abundance of mammals in the Chernobyl Exclusion Zone
Source: Sci Rep. 2020 Aug 21;10:14083. doi: 10.1038/s41598-020-70699-3 (PMC7442794; doi:10.1038/s41598-020-70699-3)
Supplement: Supplementary file 1 — Supplementary Information 1. [file 41598_2020_70699_MOESM1_ESM.docx]

Dose reconstruction supports the interpretation of decreased abundance of mammals in the Chernobyl Exclusion Zone

Karine Beaugelin-Seiller_,_  Jacqueline Garnier-Laplace, Claire Della-Vedova, Jean-Michel Métivier, Hugo Lepage, Timothy A. Mousseau and Anders Pape Møller

This text file is a three parts document. It presents at first elements on the dose reconstruction procedure (principles and equations) and secondly related data (ecological characterization of the mammals, parameter values, justification of the choice of radionuclides and soil depth to consider…). A third part is dedicated to complementary work about confounding factors and the way we dealt with them.

Two complementary supporting Excel® files are provided those contain data:

- the original data set of abundance per mammal species (Mammals CEZ-SI original dataset.xlsx)

- all the input and output data of the procedure of dose reconstruction (Mammals CEZ-SI dose reconstruction.xlsx: activities in soil and in animals, DC values, external and internal contributions to the dose rates, total dose rates and doses - all the information being detailed by species and transects; see the first datasheet “read me” for more explanation).

1. Summary of dose reconstruction procedure

Basic procedures for dose reconstruction were previously developed by Garnier-Laplace *et al.* (2015). In addition to this paper and to the information already provided in the main text, complementary explanation is provided below, related to the main principles and the associated mathematical formulation.

Radiological dose rates are additive. An exhaustive dose assessment has to include all exposure pathways for all radionuclides present, including any radioactive daughter products of significance. The calculation of total dose absorbed by a mammal species *j* due to its exposure to the radionuclide *r* depends on assessment of external and internal dose rates absorbed by this animal (respectively *EDR(j,r)* and *IDR(j,r)*in µGy h^-1^). Basically those dose rates are obtained by combining activity concentrations in soil (external contribution) or organism (internal contribution) with radionuclide-, species- and irradiation pathway-specific dose coefficients (*DCs*). As mentioned in the main text, the DC values were determined with the EDEN V3.1 software according to the ecological characteristics of each species (Tables S1 and S2). Their calculation relies on the elementary composition of each exposure medium (Table S3), taking into consideration the location of the exposed organisms relatively to its different sources of exposure, as defined by the exposure scenario (Table S4).

Internal contribution is *IDR(j,r)=DCC_int_(j,r).CR(j,r).A_soil_(r)*  (1)

Where

-*DC_int_(j,r)* radionuclide-specific dose coefficient for internal exposure of the species *j* (µGy h^-1^ per Bq kg^-1^ fresh weight, Table S8);

-*CR(j,r)* concentration ratio of the radionuclide *r* by the mammal species *j* (aggregated parameter that implicitly includes all potential exposure pathways - kg soil/ kg whole body expressed on the fresh weight basis –- see Table S5 for values);

-*A_soil_(r)* activity of the radionuclide *r* in soil (Bq kg^-1^ of soil wet weight, supporting Excel® file Mammals CEZ-SI dose reconstruction.xlsx ).

External contribution is *EDR(j,r)=DCC_on-soil_(j,r).OF(j)+DCC_soil_(j,r).(1-OF)]. A_soil_(r)*  (2)

with

-*DC_on-soil_(j,r)* dose coefficient for the species *j* externally exposed when located on soil, to the radionuclide *r* present in soil (µGy h^-1^ per Bq kg^-1^ of soil; Table S8);

-*OF(j)* occupancy factor, that is the fraction of the time that the species *j* spends on soil for a given location (dimensionless; Table S4);

-*DC_soil_(j,r)* dose coefficient for the species *j* externally exposed when located at a given distance from the soil surface to the radionuclide *r* present in soil (µGy h^-1^ per Bq kg^-1^ of soil; Table S8).

After summing on exposure pathways (external and internal contributions) the next step consists in summing on radionuclides. The total dose rate absorbed by the mammal species *j* exposed to all the radionuclides is then *TDR_j_ = Σ_r_[IDR(j,r)+EDR(j,r)]* (3).The mammal species under consideration present very heterogeneous ecological characteristics. In order to compare things with similar meaning, we decided to homogenize the ecological meaning of the species exposure by introducing a temporal dimension with a high significance in terms of population dynamics. We chose to calculate the total dose on the generation length *L_Gj_* for the mammal species *j*: *td_j_=TDR_j_* x *L_Gj_* (4)

Looking to compare transects on the basis of a single exposure criterion, the last step in our calculation consists in determining the Theoretical Total Dose on the transect *i* for the mammal species *j*: ${TTD}_{i}=\sqrt[n_{j}]{\prod_{j=1}^{n_{j}} {td}_{j}}$ (5).

1. Data

A set of different ecological data (Table S1) is needed for first DC calculation and then dose reconstruction. The dosimetric characteristics (Tables S2 and S3) were derived for mammals from the ecological data, considering an equivalent ellipsoid with a volume close (within a factor between 1 and 1.1 at maximum) to the volume calculated from the typical mass of the animal species and a tissue density of 1.07 (Table S3). Use of its environment is described for each mammal species (Table S4) whose diet was considered when it was possible to determine the best value of CR that could be applied (Table S5). The DC value depends on the depth of the contaminated soil. The final value (20 cm) was conservatively estimated from the profiles established from measurements and modelling of ^137^Cs (Figure S1) and an assessment of potential depth reached in soils by all the radionuclides characteristic of the accident deposit (Table S6). Table S7 presents a review of the knowledge related to soil contamination in the CEZ at a consistent soil depth. This suggests a huge preponderance of ^137^Cs and ^90^Sr which results in a contribution so largely dominating the dose (as illustrated for fox and moose, respectively ca 95 and 99 %; Figure S2) that it is justified to not consider other radionuclides for the complete re-analysis. DC values used for this pre-processing stage were obtained with the EDEN v3.1 software (Beaugelin-Seiller *et al*. 2006), according to the previous specifications, as the DCs for ^137^Cs, ^90^Sr and daughter elements required by the main study for the adult stage of each species (Table S7).

The original dataset is provided as an independent Excel file named Mammals CEZ-SI-original dataset.xlsx. Activity concentrations in soil, dose coefficients and all dose calculations are provided as a second independent Excel file named Mammals CEZ-SI-dose reconstruction.xlsx.

Table S1 Ecological characteristics for a typical individual of the 12 species of mammals under consideration for the Chernobyl Exclusion Zone

| Species common name  (*Latin name*) | Mass (kg) | Body length  (cm) | Home range  (km²) | Generation time* (d) | Sexual maturity (d) (F- female; M- Male) |
| --- | --- | --- | --- | --- | --- |
| Fox  (*Vulpes vulpe*s) | 6.00 | 65.0 | 5.00 | 2106 | 304 |
| Hare  (*Lepus europaeus*) | 4.00 | 55.0 | 1.50 | 730 | 236 |
| Marten  (*Martes foina*) | 1.50 | 45.0 | 1.50 | 2179 | 548 (F) - 730 (M) |
| Moose/elk  (*Alces alces*) | 350 | 250 | 65.0 | 3722 | 612 (M )- 751 (F) |
| Mouse  (*Apodemus sylvaticus*) | 0.02 | 9.50 | 0.01 | 627 | 65 (M) - 71 (F) |
| Przewalski's horse  (*Equus przewalski*) | 250 | 200 | 6.00 | 3000** | 1460 (F) – 1825 (M) |
| Red deer  (*Cervus elaphus*) | 130 | 200 | 4.00 | 5210 | 730 (M) – 852 (F) |
| Roe deer  (*Capreolus capreolus*) | 25 | 120 | 1.00 | 2336 | 413 (F) – 655 (M) |
| Squirrel  (*Sciurus vulgaris*) | 0.37 | 21.4 | 0.30 | 1613 | 296 (F) – 320 (M) |
| Stoat  (*Mustela erminea*) | 0.30 | 28.0 | 0.50 | 1079 | 95 (F) – 365 (M) |
| Wild boar  (*Sus scrofa*) | 110 | 150 | 12.0 | 2657 | 334 (F) – 768 (M) |
| Wolf  (*Lupus lupus*) | 35.0 | 100 | 400 | 2884 | 669 |

*Average age of parents of the current cohort that reflects the turnover rate of breeding individuals in a population

**no specific data, average GL value for all *Equus* species in the GL database (Pacifici *et al.* 2013).

Data sources:

Byrne ME, Webster SC, Lance SL, Love CN, Hinton TG, Shamovich D & Beasley JC (2018). Evidence of long-distance dispersal of a gray wolf from the Chernobyl Exclusion Zone. *European Journal of Wildlife Research* 64:39. DOI:10.1007/s10344-018-1201-2

Écologie Nature (2016). *Fiches Mammifères* (<http://ecologie.nature.free.fr/>)

INPN (2016). *Synthèse de données espèces*. Fiche descriptive (<https://inpn.mnhn.fr/espece/cd_nom/>)

Niethammer J & Krapp F (Eds) (2013). Handbuch der Säugertiere Europas auf DVD- Die complete Reihe. Aula Verlag, Wiebelsheim.

ONCFS (2016). *Connaître les espèces* - Présentation de la biologie et de la répartition géographique (<http://www.oncfs.gouv.fr/Connaitre-les-especes-ru73>)

Pacifici M, Santini L, Di Marco M, Baisero D, Francucci L, Grottolo Marasini G, Visconti P, Rondinini C (2013). Generation length for mammals. *Nature Conservation* 5 :87-94. DOI: 10.3897/natureconservation.5.5734

Slotta-Bachmayr L, Boegel R, Kaczensky P, Stauffer C & Walzer C (2004). Use of Population Viability Analysis to Identify Management Priorities and Success in Reintroducing Przewalski's Horses to Southwestern Mongolia. *The Journal of Wildlife Management*, 68 (4) : 790-798.

The Mammal Society (2016). *Discover Mammals*. Species Hub. Factsheet. (<http://www.mammal.org.uk/species_hub>)

Table S2 Ecological characteristics for mammal species for dosimetric calculations

|  | Body size (cm) | | | Dosimetry area^1^ |
| --- | --- | --- | --- | --- |
| Species common name  (*Latin name*) | Length | Width | Height | radius (m) |
| Fox  (*Vulpes vulpe*s) | 62 | 12 | 15 | 1400 |
| Hare  (*Lepus europaeus*) | 55 | 9.5 | 15 | 800 |
| Marten  (*Martes foina*) | 40 | 7 | 10 | 800 |
| Moose/elk  (*Alces alces*) | 240 | 37 | 77 | 5000 |
| Mouse (*Apodemus sylvaticus*) | 9.5 | 1.5 | 2.6 | 160 |
| Przewalski's horse  (*Equus przewalski*) | 200 | 47 | 52 | 1500 |
| Red deer  (*Cervus elaphus*) | 175 | 33 | 44 | 1300 |
| Roe deer  (*Capreolus capreolus*) | 110 | 17 | 26 | 700 |
| Squirrel  (*Sciurus vulgaris*) | 20 | 5 | 7 | 500 |
| Stoat  (*Mustela erminea*) | 30 | 3.9 | 5 | 500 |
| Wild boar  (*Sus scrofa*) | 140 | 33 | 46.5 | 2000 |
| Wolf  (*Lupus lupus*) | 100 | 24 | 28 | 11500 |

^1^Home range radius from Table S1 increased by the transect length (*i.e*. + 100m) and rounded to the next ten/hundred/thousand

Table S3 Elemental composition (% of the total mass), and density of media

| Element/density | Air | Animal | Soil |
| --- | --- | --- | --- |
| Al |  |  | 7.40 E+00 |
| Ar | 1.28 E+00 |  |  |
| C | 1.00 E-02 | 1.94 E+01 | 1.80 E-01 |
| Ca |  | 1.38 E+00 | 3.26 E+00 |
| Fe |  | 1.00 E-02 | 4.24 E+00 |
| H | 6.00 E-02 | 9.33 E+00 | 9.60 E-01 |
| K |  | 2.20 E-01 | 2.31 E+00 |
| Mg |  | 4.00 E-02 | 2.11 E+00 |
| N | 7.50 E+01 | 5.15 E+00 | 3.00 E-02 |
| Na |  | 2.60 E-01 | 2.39 E+00 |
| O | 2.36 E+01 | 6.29 E+01 | 5.07 E+01 |
| P |  | 6.30 E-01 | 1.10 E-01 |
| S |  | 6.40 E-01 | 1.10 E-01 |
| Si |  |  | 2.62 E+01 |
| d (g.cm^-3^) | 1.29 E-03 | 1.07 E+00 | 1.7 E+00 |

Table S4 Exposure scenarios considered by mammal species for dose reconstruction

|  | Animal at rest (burrow, nest, …) | | “Active”animal | |
| --- | --- | --- | --- | --- |
| Species common name  (*Latin name*) | Location | Occupancy factor | Location | Occupancy factor |
| Fox  (*Vulpes vulpe*s) | Burrow  (20 cm diameter, 50 cm underground) | 0.5 | Standing on soil  (body centre at 50 cm from the soil surface) | 0.5 |
| Hare  (*Lepus europaeus*) | Form  (half buried in soil) | 0.5 | On soil  (body centre at 7.5 cm from the soil surface) | 0.5 |
| Marten  (*Martes foina*) | On soil (body centre at 5 cm from the soil surface) | 0.5 | On soil  (body centre at 5 cm from the soil surface) | 0.5 |
| Moose/elk  (*Alces alces*) | On ground  (body centre at 38.5 cm from the soil surface) | 0.5 | Standing on soil  (body centre at 125 cm from the soil surface) | 0.5 |
| Mouse  (*Apodemus sylvaticus*) | Burrow  (5 cm diameter, 20 cm underground) | 0.4 | On soil  (body centre at 1.3 cm from the soil surface) | 0.6 |
| Przewalski's horse  (*Equus przewalski*) | On ground  (body centre at 26 cm from the soil surface) | 0.25 | Standing on soil  (body centre at 100 cm from the soil surface) | 0.75 |
| Red deer  (*Cervus elaphus*) | On ground  (body centre at 22 cm from the soil surface) | 0.25 | Standing on soil  (body centre at 80 cm from the soil surface) | 0.75 |
| Roe deer  (*Capreolus capreolus*) | On ground  (body centre at 13 cm from the soil surface) | 0.25 | Standing on soil  (body centre at 50 cm from the soil surface) | 0.75 |
| Squirrel  (*Sciurus vulgaris*) | In trees  (body centre at 600 cm from the soil surface) | 0.75 | On soil (body centre at 3.5 cm from the soil surface) | 0.25 |
| Stoat  (*Mustela erminea*) | Burrow (10 cm diameter, 20 cm underground) | 0.5 | Standing on soil  (body centre at 100 cm from the soil surface) | 0.5 |
| Wild boar  (*Sus scrofa*) | Wallow (half buried in soil)  On ground  (body centre at 23.25 cm from the soil surface) | 0.25  0.25 | Standing on soil  (body centre at 50 cm from the soil surface) | 0.5 |
| Wolf  (*Lupus lupus*) | Den (50 cm diameter, 100 cm underground) | 0.25 | Standing on soil  (body centre at 50 cm from the soil surface) | 0.75 |

Table S5 Concentration ratios (CRs) for internal concentration assessment of radionuclides in each of the 12 mammal species

| Species common name  (*Latin name*) | Rationale  (reference) | Am | Co | Cs | Eu^1^ | Pu | Sr | U |
| --- | --- | --- | --- | --- | --- | --- | --- | --- |
| Fox  (*Vulpes vulpe*s) | Carnivorous mammal  (IAEA 2014) | 9.80E-03 | 1.80E-01 | 1.40E-01 | 4.80E-03 | 3.10E-03 | 4.30E-01 | 3.70E-03 |
| Hare  (*Lepus europaeus*) | Herbivorous mammal  (IAEA 2014) | 1.40E-02 | 1.80E-01 | 1.50E+00 | 4.80E-03 | 9.20E-03 | 1.60E+00 | 3.70E-03 |
| Marten  (*Martes foina*) | Omnivorous mammal  (IAEA 2014) | 1.50E-02 | 1.80E-01 | 1.70E+00 | 4.80E-03 | 1.10E-02 | 1.10E+00 | 3.70E-03 |
| Moose/elk  (*Alces alces*) | Herbivorous mammal  (IAEA 2014) | 1.40E-02 | 1.80E-01 | 1.50E+00 | 4.80E-03 | 9.20E-03 | 1.60E+00 | 3.70E-03 |
| Mouse  (*Apodemus sylvaticus*) | *Apodemus flavicolis* (Beresford *et al*., 2008) | 1.50E-02 | 1.80E-01 | 1.10E+00 | 4.80E-03 | 1.10E-02 | 2.00E+00 | 3.70E-03 |
| Przewalski's horse  (*Equus przewalski*) | Herbivorous mammal  (IAEA 2014) | 1.40E-02 | 1.80E-01 | 1.50E+00 | 4.80E-03 | 9.20E-03 | 1.60E+00 | 3.70E-03 |
| Red deer  (*Cervus elaphus*) | Herbivorous mammal  (IAEA 2014) | 1.40E-02 | 1.80E-01 | 1.50E+00 | 4.80E-03 | 9.20E-03 | 1.60E+00 | 3.70E-03 |
| Roe deer  (*Capreolus capreolus*) | Herbivorous mammal  (IAEA 2014) | 1.40E-02 | 1.80E-01 | 1.50E+00 | 4.80E-03 | 9.20E-03 | 1.60E+00 | 3.70E-03 |
| Squirrel  (*Sciurus vulgaris*) | Herbivorous mammal  (IAEA 2014) | 1.40E-02 | 1.80E-01 | 1.50E+00 | 4.80E-03 | 9.20E-03 | 1.60E+00 | 3.70E-03 |
| Stoat  (*Mustela erminea*) | Carnivorous mammal  (IAEA 2014) | 9.80E-03 | 1.80E-01 | 1.40E-01 | 4.80E-03 | 3.10E-03 | 4.30E-01 | 3.70E-03 |
| Wild boar  (*Sus scrofa*) | Omnivorous mammal  (IAEA 2014) | 1.50E-02 | 1.80E-01 | 1.70E+00 | 4.80E-03 | 1.10E-02 | 1.10E+00 | 3.70E-03 |
| Wolf  (*Lupus lupus*) | Carnivorous mammal  (IAEA 2014) | 9.80E-03 | 1.80E-01 | 1.40E-01 | 4.80E-03 | 3.10E-03 | 4.30E-01 | 3.70E-03 |

^1^Chemical analogy: extrapolated from Ba data

Data sources:

Beresford NA, Gashak S, Barnett CL, Howard BJ, Chizhevsky I, Stromman G, Oughton DH, Wright SM, Maksimenko A & Copplestone D (2008). Estimating the exposure of small mammals at three sites within the Chernobyl exclusion zone – a test application of the ERICA Tool. *Journal of Environmental Radioactivity* **99**: 1496-1502

IAEA (2014). *Handbook of Parameter Values for the Prediction of Radionuclide Transfer to Wildlife*. Technical Reports Series n°479, International Atomic Energy Agency, Vienna, 228 p.

Figure S1 Distribution of ^137^Cs as measured (1988 to 1993) and predicted (2000 and 2010) for a soddy podsolic soil close to the Chernobyl NPP (Chistogalovka, 3-4 km W from the NPP; adapted from Ivanov *et al*. 1997)

Table S6 Potential maximum depth reached in a sandy soil by radionuclides deposited by the Chernobyl fallout, according to the range of their migration velocity (based on Bossew *et al*. 2004)

|  | Migration velocity (cm.y^-1^) | | Corresponding depth in soil (cm) in 2009 | |
| --- | --- | --- | --- | --- |
| Radionuclide | min | max | min | max |
| ^137^Cs | 0.141 | 0.225 | 3.2 | 5.2 |
| ^134^Cs | 0.141 | 0.234 | 3.2 | 5.4 |
| ^125^Sb | 0.148 | 0.228 | 3.4 | 5.2 |
| ^60^Co | 0.141 | 0.213 | 3.2 | 4.9 |
| ^241^Am | 0.165 | 0.242 | 3.8 | 5.6 |
| ^154^Eu | 0.169 | 0.254 | 3.9 | 5.8 |
| ^155^Eu | 0.174 | 0.257 | 4.0 | 5.9 |
| ^94^Nb | 0.156 | 0.259 | 3.6 | 6.0 |
| ^90^Sr | 0.140 | 0.203 | 3.2 | 4.7 |
| ^239/240^Pu | 0.150 | 0.197 | 3.5 | 4.5 |
| ^238^Pu | 0.151 | 0.212 | 3.5 | 4.9 |

Data sources:

Ivanov YA, Lewicky N, Levchuk SE, Prister BS, Firsakova SK, Arkhipov NP, Arkhipov AN, Kruglov SV, Alexakhin RM, Sandalls J & Askbrant S (1997). Migration of 137Cs and 90Sr from Chernobyl Fallout in Ukrainian, Belarussian and Russian soils. *Journal of Environmental Radioactivity.* **35**: 1-21.

Bossew P, Gastberger M, Gohla H, Hofer P & Hubmer A (2004). Vertical distribution of radionuclides in soil of a grassland site in Chernobyl exclusion zone. *Journal of Environmental Radioactivity* **73:** 87-99.

Table S7 Realistic extreme activity concentrations in soils (Bq.kg^-1^ in the soil layer up to 50 cm depth) for the most completely available set of radionuclides present in the CEZ, estimated for February 2009

|  | Soil activity concentration (Bq.kg^-1^) | |
| --- | --- | --- |
|  | Minimum | Maximum |
| ^137^Cs | 73.42 | 516761 |
| ^90^Sr | 39.25 | 486795 |
| ^241^Am | 0.68 | 12419 |
| ^238^Pu | 0.83 | 3763 |
| ^239^Pu | 2.43 | 8764 |
| ^240^Pu | 2.43 | 8764 |
| ^234^U | 0.95 | 20 |
| ^238^U | 1.07 | 11 |
| ^60^Co | 0.16 | 893 |
| ^154^Eu | 1.30 | 2868 |

N.B.: These data were only used to estimate the contribution of radionuclides other than Cs and Sr to the dose absorbed by mammals, to eventually limit the whole set of dose calculation to ^137^Cs and ^90^Sr when justified (see Figure S2). For this exercise, we needed to inform as exhaustively as possible the soil contamination in a single place, which was not possible from the sources mentioned in the main text for soil activities. For this we have used additional sources, cited below.

Data sources:

Chapon V, Piette L, Vesvres Mh, Coppin F, Le Marrec C, Christen R, Theodorakopoulos N, Février L, Levchuk S, Martin-Garin A, Berthomieu C & Sergeant C (2012). Microbial diversity in contaminated soils along the T22 trench of the Chernobyl experimental platform. *Applied Geochemistry* **27:** 1375–1383.

Lecomte-Pradines C., Bonzom J., Della-Vedova C., Beaugelin-Seiller K., Villenave C., Gaschak S., Coppin F., Maksimenko A., Adam-Guillermin C., Garnier-Laplace J (2014). Soil nematode assemblages as bioindicators of radiation impact in the Chernobyl Exclusion Zone. *Science of the Total Environment* **490:** 161-170.

Theodorakopoulos N (2013). Analyse de la biodiversité bactérienne d’un sol contaminé de la zone d’exclusion de Tchernobyl et caractérisation de l’interaction engagée par une souche de *Microbacterium* avec l’uranium. PhD – Université-Aix-Marseille, Ecole Doctorale ED 62 Sciences de la vie et de la santé spécialité microbiologie.

Figure S2 Illustration of the range of contribution of the 10 radionuclides measured in the CEZ soils (Table S7) to the total dose rate for the species of interest, considering the group ^137^Cs+^90^Sr (including decay products at equilibrium, as indicated by the symbol +) vs. the eight others (left: small carnivorous mammal - fox; right: large herbivorous mammal –moose/horse; below: DC values).

| DC | Internal exposure | | External exposure | | | |
| --- | --- | --- | --- | --- | --- | --- |
| µGy/h per Bq/kg | Fox | Moose/horse | On soil (moose) | In soil (fox) | Standing (fox) | Standing (moose) |
| ^137^Cs+ | 1.94E-04 | 3.06E-04 | 2.47E-05 | 3.16E-07 | 3.71E-05 | 1.15E-12 |
| ^90^Sr+ | 6.01E-04 | 6.05E-04 | 4.19E-07 | 1.04E-09 | 2.23E-08 | 1.70E-09 |
| ^241^Am | 2.95E-02 | 2.95E-02 | 5.67E-07 | 1.16E-11 | 2.20E-09 | 8.08E-12 |
| ^238^Pu | 2.95E-02 | 2.95E-02 | 3.69E-07 | 6.67E-12 | 2.43E-10 | 6.46E-12 |
| ^239^Pu | 2.77E-02 | 2.77E-02 | 3.13E-07 | 9.08E-12 | 2.35E-09 | 6.42E-12 |
| ^240^Pu | 2.78E-02 | 2.78E-02 | 3.13E-07 | 6.46E-12 | 2.32E-10 | 6.38E-12 |
| ^234^U | 2.56E-02 | 2.56E-02 | 2.44E-07 | 6.58E-12 | 1.99E-09 | 6.42E-12 |
| ^238^U | 2.25E-02 | 2.25E-02 | 1.67E-07 | 6.33E-12 | 9.71E-10 | 6.29E-12 |
| ^60^Co | 3.00E-04 | 7.88E-04 | 1.32E-04 | 4.15E-06 | 1.90E-04 | 2.00E-07 |
| ^154^Eu | 2.55E-04 | 4.96E-04 | 5.92E-05 | 1.39E-06 | 8.79E-05 | 1.75E-07 |

Table S8 DC estimates (µGy.h^-1^ per Bq.kg^-1^) per radionuclide, irradiation pathways and species (adult stage only), weighted assuming factors of 10 for α-radiation, 3 for low-β radiation (E < 10 keV), and 1 for other β-radiation and γ-radiation. The characteristics of the exposure scenes used for DC calculation are summarized in Table S4.

|  |  | Fox | Hare | Marten | Moose | Mouse | Horse | Red deer | Roe deer | Squirrel | Stoat | Wild boar | Wolf |
| --- | --- | --- | --- | --- | --- | --- | --- | --- | --- | --- | --- | --- | --- |
| Internal exposure |  |  |  |  |  |  |  |  |  |  |  |  |  |
|  | ^137^Cs+^1^ | 1.94E-04 | 1.90E-04 | 1.74E-04 | 3.06E-04 | 1.40E-04 | 2.93E-04 | 2.67E-04 | 2.23E-04 | 1.61E-04 | 1.54E-04 | 2.70E-04 | 2.40E-04 |
|  | ^90^Sr+ | 6.01E-04 | 5.97E-04 | 5.93E-04 | 6.05E-04 | 5.45E-04 | 6.05E-04 | 6.05E-04 | 6.01E-04 | 5.88E-04 | 5.80E-04 | 6.05E-04 | 6.05E-04 |
| External exposure^2^ |  |  |  |  |  |  |  |  |  |  |  |  |  |
| on soil | ^137^Cs+ |  | 8.23E-05 | 8.79E-05 | 2.47E-05 | 7.44E-05 | 2.58E-05 | 3.37E-05 | 5.14E-05 | 7.54E-05 | 9.33E-05 | 3.33E-05 |  |
|  | ^90^Sr+ |  | 7.02E-06 | 9.94E-06 | 4.19E-07 | 4.06E-05 | 6.71E-07 | 1.01E-06 | 3.08E-06 | 1.42E-05 | 1.84E-05 | 9.42E-07 |  |
| in soil | ^137^Cs+ | 3.16E-07 | 5.81E-05 |  |  | 5.45E-05 |  |  |  |  | 4.85E-05 | 3.18E-05 | 1.23E-12 |
|  | ^90^Sr+ | 1.04E-09 | 3.59E-06 |  |  | 2.84E-08 |  |  |  |  | 2.67E-08 | 9.21E-07 | 1.15E-12 |
| standing | ^137^Cs+ | 3.71E-05 |  |  | 1.15E-12 |  | 3.31E-11 | 5.41E-06 | 2.57E-05 | 3.57E-05 |  | 2.46E-05 | 9.91E-05 |
|  | ^90^Sr+ | 2.23E-08 |  |  | 1.70E-09 |  | 4.88E-09 | 1.03E-08 | 1.84E-08 | 2.25E-08 |  | 1.59E-08 | 3.24E-06 |

^1^ The symbol + indicates the inclusion of the daughter product in secular equilibrium / ^2^ for details on animal location see Table S4.

1. Complementary analysis of additional potential confounding factors

Human activities are well acknowledged to be able to negatively (i.e. use of pesticides, noise, light…) or positively (i.e. organic farming, food …) affect abundance and diversity of animals in their vicinity (Pywell, 2015; Tilman, 1999; Tuck et al, 2014).

In the areas examined for the present study, two kinds of such factors should have been considered, the spatial impact of the nuclear power plant complex itself and the existence of farms (or similar activities). We investigated the importance of overlap between the industrial area, the zone of human activities around the NPP, and the dosimetry areas of each species of mammal, as defined previously. In a second analysis, we focused on fox and characterized their numbers as a function of farming activities.

3.1. Interactions between dosimetry and industrial areas

Some of the home ranges of mammals we defined around the transects intersected the area occupied by the NPP and related activities (designed hereafter as the industrial area), effectively reducing the area available for animals. We looked to quantify this overlap for affected transects.

Given the absence of better information, the boundaries of industrial areas were manually drawn from aerial photos, taking into account all the places where there seems to be human activities associated with the NPP. We first located each sampled transect vs this area to assess the proportion of dosimetry areas that are intersected by the industrial area. For these transects, we estimated using GIS the proportion of the overlapping area with regard to the home range of each mammal. We expected to observe a higher proportion of intersected transects with an increase of the home range. The quantification of overlap was less intuitive, depending on a combination of the position of the transects and the size of home range. Table S9 summarizes our findings. As anticipated, the proportion of dosimetry areas that intersected the industrial area increases with the size of the mammal home range. For mice, which exhibit the smallest home range, there was no intersection with the industrial area. In contrast, about half of the dosimetry areas defined for wolves intersect with the industrial area. When such an intersection occurred, it represented from less than 1% (all mammals except moose/elk) up to about 10% (wild boar) of the intersected dosimetry area. Due to this low interaction, we decided not to consider the spatial impacts of the NPP as a potential cofactor in our statistical analyses.

Table S9. Characterization of the overlapping between the industrial and dosimetry areas, per mammal species

|  |  | Quantification of overlap | |
| --- | --- | --- | --- |
|  | Proportion of intersected areas | min | max |
| Fox | 28.97% | 0.05% | 2.96% |
| Hare | 22.76% | 0.03% | 1.33% |
| Marten | 22.76% | 0.03% | 1.33% |
| Moose/Elk | 29.66% | 4.04% | 4.59% |
| Mouse | 0.00% |  |  |
| Przewalski's horse | 29.66% | 0.17% | 4.90% |
| Red deer | 27.59% | 0.30% | 2.54% |
| Roe deer | 17.24% | 0.09% | 1.01% |
| Squirrel | 4.14% | 0.09% | 2.24% |
| Stoat | 10.34% | 0.01% | 1.79% |
| Wild boar | 29.66% | 0.56% | 9.77% |
| Wolf | 43.45% | 0.28% | 0.77% |

3.2. Overlapping of territorial areas of fox by farming areas or assimilated

In contrast to the potential negative effect of the presence of the NPP on the animal abundance in its immediate vicinity, commensal species of man, such as fox, takes advantage of the proximity of farmyard and other small livestock activities. The fox is an opportunistic predator that commonly feeds on poultry when possible. The number of foxes should then increase in areas where farmyards or assimilated structures exist. We attempted to test this hypothesis by investigating the relation between the number of foxes per transect and the number potentially associated with farmyards. The first step consisted of identifying in our study area such land use, which was not obvious. We opted for trying three different approaches within our immediate reach, each having pros and cons.

The first method relies on the use of the LandUse map from the OpenStreetMap (OSM) database (Planet.osm - <https://wiki.openstreetmap.org/wiki/Planet.osm>). According to OSM terminology (<https://wiki.openstreetmap.org/wiki/Tag:landuse%3Dfarmyard>), farming areas may correspond to *farmyard*, the area close to the buildings of a farm, or *farmland*, the fields located around the farmyard. Data corresponding with these two categories were extracted in relation to the study area using the tool available at <https://extract.bbbike.org/>. The file corresponding to this extraction for the 6^th^ of June 2019 is joined for any verification (Table S10). Land use data are stored there in a multi-polygons vector layer. We determined the overlap between these farming areas and the dosimetry areas for fox, as for the industrial area (see previous paragraph). For all the transects of interest, this method concluded to the absence of significant farming spaces in the dosimetry areas of foxes.

Table S10– Additional data files related to spatial analyses

| GIS data needed by method-1 | Satellite images analysed for method-3 |
| --- | --- |
|  |  |

The second approach applied was based on satellite imagery (Esri World Imagery 2019^^[[1]](#footnote-1)^^). Residential areas appeared from these images largely associated with farmland, suggesting the possibility of livestock for domestic use such as poultry. This would constitute a point of attraction for fox. Overlap between these areas and the dosimetry areas of fox was estimated (Table S10, method 2).

Only transects located in sector D (see map in the main text) exhibited a significant overlap (from ca 20 to 35%) between such residential areas and dosimetry areas of fox. Plotting the change in fox number with regard to the increase of the presence of farming activities in their dosimetry area did not show any significant trend (Figure S3).

A deeper analysis of the images of residential areas revealed they were somewhat heterogeneous, and included significant areas of forest or farmland. A restricted residential area was defined and manually drawn (Table S10), excluding forests and farmland. This method reduced by about a factor 2 the residential area in sector D, leading to an overlap varying from 8 to 16%. Elsewhere, the overlap slightly increased but remained lower than 7.5%. There was still no significant relationship between the number of foxes and the presence of farming activities in their dosimetry areas (Figure S3).

All three methods tested had weaknesses and were highly uncertain. They relied however on independent data sources and interpretation, and none suggested any obvious link between potential farming activities and the number of foxes. We decided not to include the presence of farming activities in our statistical analyses.

3.3. Uncertainties

Dealing with interactions between mammal abundance and human activity first needs the definition of areas where such activities may take place. Regarding industrial activity in the CEZ, that means to demarcate the area around the reactors occupied for their functioning and maintenance. The boundaries of this area were more or less unchanged with time, the location of the NPP is well known, there is neither temporal nor spatial problems to solve. The main uncertainty about the area definition comes from the spatial resolution of aerial photos used for that.

At the opposite, defining farming areas in the CEZ was really challenging when looking to localize poultry farming. Being an exclusion zone, the CEZ is today not supposed to welcome too many farming activities that are one of the common categories of land use. The first source of information on land use is related atlas and maps, some being easily and freely available. The map we used distinguished *farmyard* and *farmland* areas, both presenting information we gathered. The representativeness of these categories with regard to poultry farming is totally unknown. To overcome this limitation a cross-checking was attempted with satellite imagery. The interpretation of such images is highly subjective, with regard to the nature and the boundaries of the targeted areas. Additionally, there was no temporal concordance between the census and the acquisition of land use information.

3.4. Conclusions

It is generally assumed that there is the potential for human activities to influence the presence of mammals in the CEZ. Among these activities, two appeared as potentially significant, the industrial activities around the NPP and the farming activities almost everywhere in our study area. For both cases, the available data we collected and the methods we applied did not reveal sufficient interaction potential to justify the inclusion of these activities as confounding factors in our statistical analyses.

Table S.11 – Percentage of overlap between dosimetry areas of foxes and farming areas (farmyard and residential places (method_2); manually selected residential (method_3) for intersected transects.

| Origins of the transect* | | Percent of overlap | | Number of foxes as counted in snow |
| --- | --- | --- | --- | --- |
| Latitude | Longitude | Method_2 | Method_3 |  |
| 51.39108333 | 30.06416667 | 1.0% | 0.0% | 1 |
| 51.39106667 | 30.06303333 | 1.0% | 0.0% | 1 |
| 51.39046667 | 30.06291667 | 1.0% | 0.0% | 1 |
| 51.38973333 | 30.06303333 | 1.0% | 0.0% | 1 |
| 51.38893333 | 30.06295 | 1.0% | 0.0% | 1 |
| 51.38808333 | 30.06295 | 1.0% | 0.0% | 1 |
| 51.38756667 | 30.06226667 | 1.0% | 0.0% | 0 |
| 51.38691667 | 30.06273333 | 1.0% | 0.0% | 0 |
| 51.38631667 | 30.06335 | 1.0% | 0.0% | 0 |
| 51.3819 | 30.0631 | 1.0% | 0.0% | 0 |
| 51.38253333 | 30.06318333 | 1.0% | 0.0% | 0 |
| 51.38128333 | 30.06478333 | 1.0% | 0.0% | 0 |
| 51.39105 | 30.06541667 | 1.0% | 0.0% | 2 |
| 51.39121667 | 30.06666667 | 1.0% | 0.0% | 2 |
| 51.39126667 | 30.0513 | 0.2% | 0.0% | 2 |
| 51.39133333 | 30.06918333 | 1.0% | 0.0% | 1 |
| 51.3914 | 30.07045 | 1.0% | 0.0% | 1 |
| 51.39068333 | 30.07046667 | 1.0% | 0.0% | 0 |
| 51.38988333 | 30.07038333 | 1.0% | 0.0% | 0 |
| 51.38938333 | 30.07088333 | 1.0% | 0.0% | 0 |
| 51.38915 | 30.07191667 | 1.0% | 0.0% | 1 |
| 51.38838333 | 30.07213333 | 1.0% | 0.0% | 0 |
| 51.38765 | 30.07236667 | 1.0% | 0.0% | 1 |
| 51.38691667 | 30.07225 | 1.0% | 0.0% | 1 |
| 51.38615 | 30.07218333 | 1.0% | 0.0% | 0 |
| 51.38538333 | 30.07223333 | 1.0% | 0.0% | 0 |
| 51.38478333 | 30.07195 | 1.0% | 0.0% | 0 |
| 51.38515 | 30.07093333 | 1.0% | 0.0% | 0 |
| 51.38561667 | 30.06996667 | 1.0% | 0.0% | 0 |
| 51.38606667 | 30.06903333 | 1.0% | 0.0% | 1 |
| 51.38661667 | 30.06966667 | 1.0% | 0.0% | 0 |
| 51.38735 | 30.0697 | 1.0% | 0.0% | 0 |
| 51.38811667 | 30.06976667 | 1.0% | 0.0% | 0 |
| 51.38855 | 30.06903333 | 1.0% | 0.0% | 0 |
| 51.38891667 | 30.06791667 | 1.0% | 0.0% | 0 |
| 51.38908333 | 30.06678333 | 1.0% | 0.0% | 0 |
| 51.38948333 | 30.05578333 | 1.0% | 0.0% | 0 |
| 51.3898 | 30.0648 | 1.0% | 0.0% | 2 |
| 51.39021667 | 30.06385 | 1.0% | 0.0% | 0 |
| 51.39041667 | 30.06296667 | 1.0% | 0.0% | 1 |
| 51.39023333 | 30.064 | 1.0% | 0.0% | 1 |
| 51.39058333 | 30.06526667 | 1.0% | 0.0% | 1 |
| 51.3908 | 30.06616667 | 1.0% | 0.0% | 0 |
| 51.11383333 | 30.13663333 | 32.3% | 15.0% | 3 |
| 51.11301667 | 30.13656667 | **34.0%** | **15.9%** | 1 |
| 51.11328333 | 30.13636667 | 33.3% | 15.5% | 1 |
| 51.11343333 | 30.13541667 | 32.1% | 15.0% | 1 |
| 51.11381667 | 30.13443333 | 30.4% | 13.9% | 1 |
| 51.1139 | 30.13328333 | 28.9% | 12.9% | 2 |
| 51.11378333 | 30.13205 | 27.6% | 12.0% | 2 |
| 51.10546667 | 30.13095 | 27.8% | 12.6% | 2 |
| 51.11375 | 30.12981667 | 24.5% | 10.3% | 2 |
| 51.11481667 | 30.12993333 | 22.6% | 9.2% | 1 |
| 51.11548333 | 30.12955 | 20.6% | 8.1% | 0 |
| 51.11523333 | 30.13075 | 22.7% | 9.2% | 1 |
| 51.11486667 | 30.13185 | 24.9% | 10.5% | 1 |
| 51.1146 | 30.13348333 | 27.6% | 12.2% | 2 |
| 51.11505 | 30.13421667 | 27.3% | 11.9% | 2 |
| 51.11526667 | 30.13516667 | 27.6% | 12.1% | 1 |
| 51.11513333 | 30.13625 | 28.8% | 12.8% | 1 |
| 51.11518333 | 30.13726667 | 29.3% | 13.1% | 1 |
| 51.11495 | 30.13746667 | 30.1% | 13.6% | 0 |
| 51.1143 | 30.13701667 | 31.5% | 14.4% | 0 |
| 51.11375 | 30.13845 | 33.9% | 15.4% | 1 |
| 51.11411667 | 30.13853333 | 33.0% | 14.9% | 1 |
| 51.10333333 | 30.02355 | 1.0% | 2.6% | 2 |
| 51.10396667 | 30.02383333 | 2.0% | 3.4% | 1 |
| 51.10473333 | 30.02386667 | 3.1% | 4.8% | 1 |
| 51.1055 | 30.02353333 | 3.2% | 5.7% | 1 |
| 51.10616667 | 30.02293333 | 3.4% | 6.1% | 1 |
| 51.10678333 | 30.0224 | 3.7% | 6.4% | 1 |
| 51.10743333 | 30.02201667 | 4.0% | 6.7% | 1 |
| 51.10801667 | 30.02126667 | 4.3% | 7.0% | 1 |
| 51.10853333 | 30.0206 | 4.5% | 7.3% | 2 |
| 51.10828333 | 30.01951667 | 4.5% | 7.2% | 1 |
| 51.10796667 | 30.0185 | 4.4% | 7.1% | 2 |
| 51.10751667 | 30.01765 | 4.2% | 6.6% | 1 |
| 51.10693333 | 30.01685 | 3.7% | 5.8% | 1 |
| 51.10591667 | 30.01536667 | 2.4% | 4.2% | 1 |
| 51.10535 | 30.01473333 | 1.8% | 3.4% | 1 |
| 51.10478333 | 30.01405 | 1.5% | 2.8% | 2 |
| 51.10413333 | 30.01356667 | 1.2% | 2.2% | 1 |
| 51.10476667 | 30.01391667 | 1.5% | 2.7% | 1 |
| 51.10333333 | 30.02235 | 1.0% | 2.5% | 1 |
| 51.10326667 | 30.02193333 | 1.0% | 2.5% | 1 |

* transects presented are only those for which at least one of the two methods give a percentage of overlap higher than 0


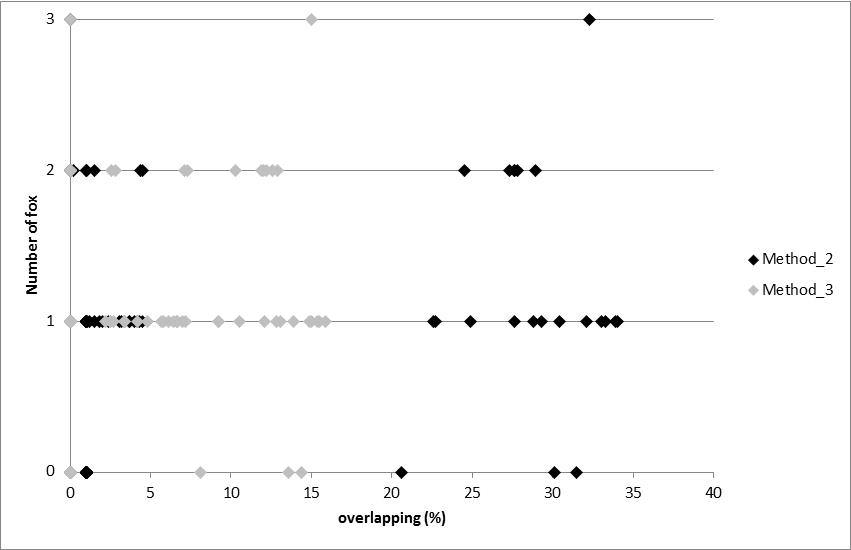


Overlap (%)

No. foxes

Figure S.3 Change in number of foxes with increasing surface of farming areas in their dosimetry areas

References

Pywell, T.F., Heard, M.S., Woodcock, B.A., Hinsley, S., Ridding, L., Nowakowski, M., Bullock, J.M. (2015). Wildlife-friendly farming increases crop yield: evidence for ecological intensification. *Proceedings of the Royal Society – B Biological Sciences* **282**: 20151740.

Tilman, D. (1999). Global environmental impacts of agricultural expansion: The need for sustainable and efficient practices. *Proceedings of the National Academy of Sciences of the United States of America* **96** (11) 5995-6000.

Tuck, S.L., Winqvist,C., Mota,F., Ahnström,J. Turnbull, L.A. & Bengtsson, J (2014). Land-use intensity and the effects of organic farming on biodiversity: a hierarchical meta-analysis. *Journal of Applied Ecology* **51**(3): 746-755.

1. Pictures used for this study (High Resolution 30cm Imagery) were obtained from the ESRI database World Imagery (<https://services.arcgisonline.com/ArcGIS/rest/services/World_Imagery/MapServer>), which contains high-resolution satellite and aerial imagery, typically within 3-5 years of currency, from multiple sources (Esri, DigitalGlobe, Earthstar Geographics, CNES/Airbus DS, GeoEye, USDA FSA, USGS, Aerogrid, IGN, IGP, and the GIS User Community). The satellite images used for the Chernobyl NPP area were taken in 2017 (DigitalGlobe; no older image available) while the images of the rest of the studied area (including sector C and D) were taken between 2011 and 2013 (DigitalGlobe). The ground resolution of the source data is 0.5 meters and the objects in map are within 8.5 meters of the true location. [↑](#footnote-ref-1)
